# Multiplexing Gains under Mixed-Delay Constraints on Wyner’s Soft-Handoff Model

**DOI:** 10.3390/e22020182

**Published:** 2020-02-05

**Authors:** Homa Nikbakht, Michèle Angela Wigger, Shlomo Shamai (Shitz)

**Affiliations:** 1 Laboratoire Traitement et Communication de l’Information (LTCI), Telecom Paris, Institut polytechnique de Paris, 91120 Palaiseau, France; 2 Department of Electrical Engineering, Technion–Israel Institute of Technology, Haifa 32000, Israel

**Keywords:** mixed-delay constraints, multiplexing gain region, Wyner’s soft-handoff model

## Abstract

This paper analyzes the multiplexing gains (MG) achievable over Wyner’s soft-handoff model under mixed-delay constraints, that is, when delay-sensitive and delay-tolerant data are simultaneously transmitted over the network. In the considered model, delay-sensitive data cannot participate or profit in any ways from transmitter or receiver cooperation, but delay-tolerant data can. Cooperation for delay-tolerant data takes place over rate-limited links and is limited to a fixed number of cooperation rounds. For the described setup, inner and outer bounds are derived on the set of MG pairs that are simultaneously achievable for delay-sensitive and delay-tolerant data. The bounds are tight in special cases and allow us to obtain the following conclusions. For large cooperation rates, and when both transmitters and receivers can cooperate, it is possible to simultaneously attain maximum MG for delay-sensitive messages and maximum sum MG for all messages. For comparison, in scheduling schemes (also called time-sharing schemes), the largest achievable sum MG decreases linearly with the MG of delay-sensitive messages. A similar linear decrease is proved for any coding scheme, not only for scheduling schemes, if only transmitters or only receivers can cooperate (but not both) and if delay-sensitive messages have moderate MG. In contrast, if the MG of delay-sensitive messages is small, the maximum sum MG can be achieved even with only transmitter or only receiver cooperation. To summarise, when cooperation rates are high and both transmitters and receivers can cooperate or when delay-sensitive messages have small MG, then transmitting delay-sensitive messages causes no penalty on the sum-MG. In other regimes, this penalty increases proportionally to the delay-tolerant MG in the sense that increasing the delay-sensitive MG by Δ penalises the largest achievable delay-tolerant MG by 2Δ and thus the sum MG by Δ.

## 1. Introduction

One of the major challenges of today’s wireless communication networks is to design coding schemes for transmission of heterogeneous traffic types. For example, different data streams (pertaining to different applications) can be subject to different delay constraints. Such mixed delay constraints in wireless networks have recently been studied in References [1,2,3,4,5]. In particular, Reference [1] proposes a broadcasting approach over a single-antenna fading channel to communicate a stream of “fast” messages, which have to be sent over a single coherence block, and a stream of “slow” messages, which can be sent over multiple blocks. A similar approach was taken in Reference [2] but for a broadcast scenario with *K* users. Instead of superposing “slow” on “fast” messages, this latter work proposes a scheduling approach to give preference to the communication of “fast” messages. A scheduling algorithm that prioritizes “fast” messages over “slow” messages was also proposed in Reference [3]. In particular, “fast” messages can be stored in the buffer for only one scheduling period.

A related scenario was introduced in Reference [4] for cloud radio access networks (C-RAN). In Reference [4], the messages sent by the mobile users close to the base stations (BS) are directly decoded at these BSs, whereas messages from users located further away are decoded at the cloud processor. In our terminology, the messages sent by close by users are “fast” messages because they will incur smaller decoding delay, and the messages sent by further away users are “slow” messages because their decoding is performed at the cloud processor and thus takes more time. In the spirit of this interpretation, we considered a similar approach in Reference [5], but where in our setup each user can send both “fast” and “slow” messages and “fast” messages have to be decoded immediately at the BS, whereas “slow” messages are decoded at the central processor. Moreover, in Reference [5] the channel from the mobile users to the BSs is modelled as a fading channel. The results in Reference [5] show that for small front haul capacities from the BSs to the cloud processor it is beneficial, in terms of sum-rate, to send both “fast” and “slow” messages. However, when the rate of “fast” messages is already large, then increasing it further, deteriorates the sum-rate of the system. In this regime, the stringent delay constraints on the “fast” messages penalise the overall performance.

In this paper, we consider a cellular network without a cloud processor, but where neighbouring BS or/and neighbouring mobile users can cooperate over dedicated *cooperation links* that do not interfere with the main communications channel. These cooperation links can model the backhauls between BSs or bluetooth or microwave links between neighbouring mobiles. We consider a scenario in which the transmitters have both delay-sensitive and delay-tolerant messages to transmit to their corresponding receivers. In our setup, delay-sensitive messages, the “fast” messages, cannot profit from cooperation due to their stringent delay constraints. That means they cannot participate in the transmitter (Tx) cooperation phase and they also have to be decoded prior to the receiver (Rx) cooperation phase. Delay-tolerant messages, the “slow” messages, can profit from both Tx- and Rx-cooperation. This specific problem formulation has strong similarity with (and was also inspired by) the model studied by Huleihel and Steinberg [6,7] where cooperation links may be absent and some of the messages are only sent if these links are present.

The focus of this paper is on the pairs of *Multiplexing Gains (MG)*, also called degree of freedom or capacity prelog, that are simultaneously achievable for the “fast” and “slow” messages in our setup. We consider Wyner’s soft-handoff model also known as one-dimensional Wyner model [8,9]. Notice that cooperation in this network has been studied in various works including [10,11,12,13,14,15,16,17]. The focus of [10] is to identify associations between Rxs and Txs, that maximize the average MG across both uplink and downlink sessions, using cooperative transmission and reception schemes between Txs. More closely related are the works in [11,12]. In particular [12] is related to our setup when only “slow” messages are transmitted. In [12], neighbouring Txs can cooperate with each other over a fixed number of rounds and neighbouring Rxs can cooperate with each other over a fixed number of rounds. It is proved that for small cooperation prelogs, a single cooperation round at the Txs or at the Rxs achieves the same MG as when the number of cooperation rounds is unlimited. On the other hand, for large cooperation prelogs, the maximum per user MG increases with every additional cooperation round that is permitted either at the Txs or at the Rxs.

In the present work, we thus extend the work in Reference [12] to accommodate not only “slow” messages that can tolerate the delays from cooperation, but also “fast” messages that have to be encoded and decoded without further delay and thus cannot profit from cooperation. Notice that the standard approach to combine the transmissions of delay-tolerant and delay-sensitive data is to apply a smart scheduling algorithm and thus to time-share a scheme for only delay-tolerant data with a scheme for only delay-sensitive data. Since the maximum MG attained for only delay-tolerant data is larger (there are less constraints imposed on this transmission) than the maximum MG attained for only delay-sensitive data, this approach can achieve the maximum sum-MG only when exclusively sending delay-tolerant data. More specifically, for scheduling schemes, the sum-MG decreases linearly with the MG of delay-sensitive data. In this paper, we determine the set of all achievable delay-sensitive and delay-tolerant MG pairs, that is, the *optimal MG region*, in the function of the prelogs of the cooperation links and the total number of cooperation rounds allowed for “slow” messages. The obtained results show that (for Wyner’s soft-handoff model) when only Txs or only Rxs can cooperate, transmitting “fast” messages at low MG does not penalise the sum-MG of “slow” and “fast” messages. In contrast, when the MG of “fast” messages is large, this is not the case and increasing the MG of “fast” messages by Δ comes at the expense of decreasing the MG of “slow” messages by 2Δ and the sum MG by Δ. When the cooperation rates are sufficiently large and both Txs and Rxs can cooperate, then it is possible to accommodate the largest possible MG for delay-sensitive messages without decreasing the maximum sum-MG. The stringent delay constraints thus do not harm the overall performance in this scenario.

To achieve the described performance, we propose a new coding scheme where every second Tx sends a “fast” message and the other Txs send a “slow” message or no message at all. Due to the structure of Wyner’s soft-handoff network, communication of “fast” messages is only interfered by transmissions of “slow” messages. This interference can thus be described during the Tx-cooperation phase and precanceled at the Txs sending the “fast” messages. On the other hand, Rxs that have to decode “fast” messages do this without further delay and describe their decoded messages during the Rx-cooperation phase to their adjacent Rxs. Since we alternated the transmission of “fast” and “slow” messages across Tx/Rx-pairs, these adjacent Rxs decode “slow” messages. With the obtained cooperation messages they can thus first subtract the interference from the “fast” messages and then decode their own “slow” messages. The described mechanism allows interference-free transmission of “fast” messages to be accommodated on every second Tx/Rx pair without disturbing the transmission of “slow” messages. Employing an optimal coding scheme for the transmission of “slow” messages on all other Tx/Rx pairs will then give the same over-all performance as when using an optimal coding scheme to send a “slow” message on each and every Tx/Rx pair. This explains why with Tx- and Rx-cooperation the maximum MG can be attained even with a “fast” MG of L/2, where L denotes the number of antennas at each Tx and Rx. Notice that this is the largest MG when only “fast” messages but no “slow” messages are transmitted.

### 1.1. Organization

The rest of this paper is organised as follows. We end this section with some remarks on notation. The following Section 2 describes the problem setup. Section 3 presents our results when only transmitters or only receivers can cooperate and Section 4 the results when transmitters *and* receivers can cooperate. Section 5 concludes the main body of the paper. Technical proofs of the converse results are referred to in appendices.

### 1.2. Notation

We use the shorthand notations “Rx“ for “Receiver“ and “Tx“ for “Transmitter“. The set of all integers is denoted by Z, the set of positive integers by $Z^{+}$ and the set of real numbers by R. For other sets we use calligraphic letters, for example, X. Random variables are denoted by uppercase letters, for example, *X*, and their realizations by lowercase letters, for example, *x*. For vectors we use boldface notation, that is, upper case boldface letters such as X for random vectors and lower case boldface letters such as x for deterministic vectors.) Matrices are depicted with sans serif font, for example, H. We also write $X^{n}$ for the tuple of random variables $\left(X_{1},\ldots ,X_{n}\right)$ and $X^{n}$ for the tuple of random vectors $\left(X_{1},\ldots ,X_{n}\right)$.

## 2. Problem Description

**Figure 1:**
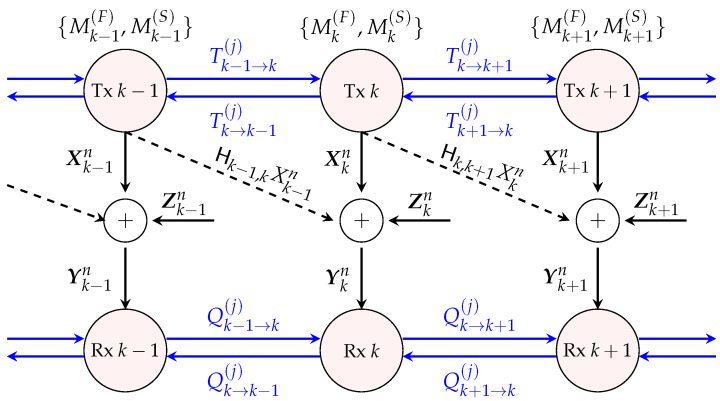
System model with transmitter (Tx-) and receiver (Rx-)cooperation.

Consider Wyner’s soft-handoff network with *K* Txs and *K* Rxs that are aligned on two parallel lines so that each Tx *k* has two neighbours, Tx k−1 and Tx k+1, and each Rx *k* has two neighbours, Rx k−1 and Rx k+1. Interference is short-range in the sense that the signal sent by Tx *k* is observed only by Rx *k* and by the neighbouring Rx k+1 (see Figure 1). Let Txs and Rxs be equipped with L>0 antennas each. The time-*t* channel output at Rx *k* is then described as

(1) $$Y_{k,t}=H_{k,k}X_{k,t}+H_{k-1,k}X_{k-1,t}+Z_{k,t},$$

where $X_{k,t}$ and $X_{k-1,t}$ are the real L-dimensional vectors sent by Tx *k* and Tx k−1 at time *t*; $\left\{Z_{k,t}\right\}$ is a noise sequence consisting of i.i.d. standard Gaussian vectors; $H_{k,k}$ and $H_{k-1,k}$ are fixed full rank channel matrices; and $X_{0,t}=0$ for all *t*.

Each Tx k∈{1,…,K} wishes to send a pair of independent messages $M_{k}^{\left(F\right)}$ and $M_{k}^{\left(S\right)}$ to Rx *k*. The “fast” message $M_{k}^{\left(F\right)}$ is uniformly distributed over the set $M_{k}^{\left(F\right)}≜\left\{1,\ldots ,\left\lfloor 2^{nR_{k}^{\left(F\right)}}\right\rfloor \right\}$ and needs to be decoded subject to a stringent delay constraint, as we explain shortly. The “slow” message $M_{k}^{\left(S\right)}$ is uniformly distributed over $M_{k}^{\left(S\right)}≜\left\{1,\ldots ,\left\lfloor 2^{nR_{k}^{\left(S\right)}}\right\rfloor \right\}$ and is subject to a less stringent decoding delay constraint. Here, *n* denotes the blocklength of transmission and $R_{k}^{\left(F\right)}$ and $R_{k}^{\left(S\right)}$ the rates of transmissions of the “fast” and “slow” messages.

We consider three different cooperation scenarios:

1. Neighbouring Txs cooperate by communicating during $D_{\mathrm{Tx}}>0$ rounds over dedicated cooperation links. Rxs cannot cooperate, and so the number of Rx-cooperation rounds is $D_{\mathrm{Rx}}=0$. (This scenario is termed “Tx-cooperation Only“)
2. Neighbouring Rxs cooperate by communicating during $D_{\mathrm{Rx}}>0$ rounds over dedicated cooperation links. Txs cannot cooperate, and so the number of Tx-cooperation rounds is $D_{\mathrm{Tx}}=0$. (Termed “Rx-cooperation Only“)
3. Neighbouring Txs cooperate during $D_{\mathrm{Tx}}>0$ rounds over dedicated cooperation links and neighbouring Rxs cooperate during $D_{\mathrm{Rx}}>0$ rounds. (Termed “Tx- and Rx-cooperation“).

The cooperative communication is subject to a total delay constraint

(2) $$D_{\mathrm{Tx}}+D_{\mathrm{Rx}}\leq D,$$

where D>0 is a given parameter of the system. In the “Tx-cooperation Only” scenario $D_{\mathrm{Rx}}$ has to be 0 and thus $D_{\mathrm{Tx}}\leq D$. Similarly in the “Rx-cooperation Only” scenario, $D_{\mathrm{Tx}}=0$ and $D_{\mathrm{Rx}}\leq D$. For “Tx- and Rx-cooperation” the values of $D_{\mathrm{Tx}}$ and $D_{\mathrm{Rx}}$ are design parameters and can be chosen arbitrary such that (2) is satisfied. As we will see, in our setup the cooperative communication only concerns “slow” messages, because “fast” messages are subject to stringent delay constraint and thus have to be transmitted and decoded without further delay.

We describe the encoding at the Txs. In the case of Tx-cooperation, neighbouring Txs can communicate to each other over dedicated noise-free, but rate-limited, links. Communication takes place over $D_{\mathrm{Tx}}>0$ rounds and can depend only on the “slow” messages but not on the “fast” messages. In each cooperation round $j\in \{1,\ldots ,D_{\mathrm{Tx}}\}$, Tx *k* produces a cooperation message $T_{k\to \ell }^{\left(j\right)}$ for each of its neighbours ℓ∈{k−1,k+1} by computing

(3) $$T_{k\to \ell }^{\left(j\right)}=\xi_{k\to \ell }^{\left(n\right)}\left(M_{k}^{\left(S\right)},{\left\{T_{\ell^{\prime }\to k}^{\left(1\right)},\ldots ,T_{\ell^{\prime }\to k}^{\left(j-1\right)}\right\}}_{\ell^{\prime }\in \left\{k-1,k+1\right\}}\right),\;j\in \left\{1,\ldots ,D_{\mathrm{Tx}}\right\},\;\ell \in \left\{k-1,k+1\right\},$$

for some function $\xi_{k\to \ell }^{\left(n\right)}$ on appropriate domains. Tx *k* sends the messages $T_{k\to \ell }^{\left(1\right)},\ldots ,T_{k\to \ell }^{\left(D_{\mathrm{Tx}}\right)}$ over the cooperation link to Tx ℓ∈{k−1,k+1}. The rate-limitation on the cooperation link imposes

(4) $$\sum_{j=1}^{D_{\mathrm{Tx}}}H\left(T_{k\to \ell }^{\left(j\right)}\right)\leq \mu_{Tx}\cdot \frac{n}{2}\log\left(P\right),\;k\in \left\{1,\ldots ,K\right\},\;\ell \in \left\{k-1,k+1\right\},$$

for a given $\mu_{\mathrm{Tx}}>0$.

Tx *k* finally computes its channel inputs $X_{k}^{n}=\left(X_{k,1},\ldots ,X_{k,n}\right)\in R^{L\times n}$ as a function of its “fast” and “slow” messages and of all the $2D_{\mathrm{Tx}}$ cooperation messages that it obtained from its neighbouring transmitters:

(5) $$X_{k}^{n}={\tilde{f}}_{k}^{\left(n\right)}\left(M_{k}^{\left(F\right)},M_{k}^{\left(S\right)},{\left\{T_{\ell^{\prime }\to k}^{\left(1\right)},\ldots ,T_{\ell^{\prime }\to k}^{\left(D_{\mathrm{Tx}}\right)}\right\}}_{\ell^{\prime }\in \left\{k-1,k+1\right\}}\right).$$

In the setup without Tx-cooperation, Tx *k* computes its channel inputs $X_{k}^{n}$ simply as a function of its “fast” and “slow” messages:

(6) $$X_{k}^{n}=f_{k}^{\left(n\right)}\left(M_{k}^{\left(F\right)},M_{k}^{\left(S\right)}\right).$$

In any case (i.e., with and without Tx-cooperation), the channel inputs have to satisfy the average block-power constraint

(7) $$\frac{1}{n}\sum_{t=1}^{n}\left|\right|X_{k,t}{\left|\right|}^{2}\leq P,\;\forall \;k\in \left\{1,\ldots ,K\right\},$$

almost surely.

We now describe the decoding. In the case of Rx-cooperation, decoding takes place in two phases. During the first *fast-decoding phase*, each Rx *k* decodes its intended “fast” message $M_{k}^{\left(F\right)}$ based on its own channel outputs $Y_{k}^{n}=\left(Y_{k,1},\ldots ,Y_{k,n}\right)\in R^{L\times n}$. So, it produces:

(8) $${\hat{M}}_{k}^{\left(F\right)}=g_{k}^{\left(n\right)}\left(Y_{k}^{n}\right),$$

where $g_{k}^{\left(n\right)}$ denotes a decoding function on appropriate domains.

In the subsequent *slow-decoding phase*, Rxs first communicate with their neighbours during $D_{\mathrm{Rx}}\geq 0$ rounds over dedicated noise-free and rate-limited links, and then they decode their intended “slow” messages based on their outputs and based on this exchanged information. Specifically, in each cooperation round $j\in \{1,\ldots ,D_{\mathrm{Rx}}\}$, each Rx *k*, for k∈{1,…,K}, produces a cooperation message $Q_{k\to \ell }^{\left(j\right)}$ for each of its neighbours ℓ∈{k−1,k+1}:

(9) $$Q_{k\to \ell }^{\left(j\right)}=\psi_{k,\ell }^{\left(n\right)}\left(Y_{k}^{n},\left\{Q_{\ell^{\prime }\to k}^{\left(1\right)},\ldots ,Q_{\ell^{\prime }\to k}^{\left(j-1\right)}{\}}_{\ell^{\prime }\in \left\{k-1,k+1\right\}}\right\}\right),$$

for an encoding function $\psi_{k,\ell }^{\left(n\right)}$ on appropriate domains. Rx *k* then sends the messages $Q_{k\to \ell }^{\left(1\right)},\ldots ,$$Q_{k\to \ell }^{\left(D_{\mathrm{Rx}}\right)}$ over the cooperation link to Rx ℓ∈{k−1,k+1}. The rate-limitation on the cooperation link imposes

(10) $$\sum_{j=1}^{D_{\mathrm{Rx}}}H\left(Q_{k\to \ell }^{\left(j\right)}\right)\leq \mu_{Rx}\cdot \frac{n}{2}\log\left(P\right),\;k\in \left\{1,\ldots ,K\right\},\;\ell \in \left\{k-1,k+1\right\},$$

for some given $\mu_{\mathrm{Rx}}>0$.

After the last cooperation round, each Rx *k* decodes its desired “slow” messages as

(11) $${\hat{M}}_{k}^{\left(S\right)}=b_{k}^{\left(n\right)}\left(Y_{k}^{n},{\left\{Q_{\ell^{\prime }\to k}^{\left(1\right)},\ldots ,Q_{\ell^{\prime }\to k}^{\left(D_{\mathrm{Rx}}\right)}\right\}}_{\ell^{\prime }\in \left\{k-1,k+1\right\}}\right),$$

where $b_{k}^{\left(n\right)}$ denotes a decoding function on appropriate domains.

For each of the three cooperation scenarios, given cooperation prelogs $\mu_{\mathrm{Rx}},\mu_{\mathrm{Tx}}\geq 0$ and maximum delay D, a MG pair $\left(S^{\left(F\right)},S^{\left(S\right)}\right)$ is called *achievable*, if for every positive integer *K* there exists a sequence of average rates ${\left\{R_{K}^{\left(F\right)}\left(P\right),R_{K}^{\left(S\right)}\left(P\right)\right\}}_{P>0}$ so that

(12) $$\begin{array}{cc} S^{\left(F\right)} & ≜\underset{K\to \infty }{\bar{\lim}}\;\underset{P\to \infty }{\bar{\lim}}\;\frac{R_{K}^{\left(F\right)}\left(P\right)}{\frac{1}{2}\log\left(P\right)}, \end{array}$$

(13) $$\begin{array}{cc} S^{\left(S\right)} & ≜\underset{K\to \infty }{\bar{\lim}}\;\underset{P\to \infty }{\bar{\lim}}\;\frac{R_{K}^{\left(S\right)}\left(P\right)}{\frac{1}{2}\log\left(P\right)}, \end{array}$$

and so that for each average rate pair $\left(R_{K}^{\left(F\right)}\left(P\right),R_{K}^{\left(S\right)}\left(P\right)\right)$ it is possible to find a set (in the blocklength *n*) of encoding, cooperation, and decoding functions satisfying constraints (2), (4), (7), and (10) and with vanishing probability of error:

(14) $$p\left(\mathrm{error}\right)≜P\left[\underset{k\in \{1,\ldots ,K\}}{⋃}\left(\left({\hat{M}}_{k}^{\left(F\right)}\neq M_{k}^{\left(F\right)}\right)\cup \left({\hat{M}}_{k}^{\left(S\right)}\neq M_{k}^{\left(S\right)}\right)\right)\right]\to 0\;\mathrm{as}\;n\to \infty .$$

The closure of the set of all achievable MG pairs $\left(S^{\left(F\right)},S^{\left(S\right)}\right)$ is called *optimal MG region*. In the case of Tx-cooperation only, it is denoted $S_{\mathrm{Tx}}^{🟉}\left(\mu_{\mathrm{Tx}},D\right)$, in the case of Rx-cooperation only $S_{\mathrm{Rx}}^{🟉}\left(\mu_{\mathrm{Rx}},D\right)$, and in the case of Tx- and Rx-cooperation $S^{🟉}\left(\mu_{\mathrm{Tx}},\mu_{\mathrm{Rx}},D\right)$.

## 3. Rx- or Tx-Cooperation Only

In the following two subsections, we consider the Rx-cooperation only scenario and the Tx-cooperation only scenario. For each scenario we present coding schemes and the optimal MG region. The scenario with both Tx-and Rx-cooperation is treated in the next Section 4.

### 3.1. Optimal MG Region and Coding Schemes for Rx-Cooperation Only

**Theorem** **1** (Optimal Multiplexing Gain Region: Rx-cooperation Only)**.** *For any given $\mu_{\mathrm{Rx}}>0$, the MG region $S_{\mathrm{Rx}}^{🟉}\left(\mu_{\mathrm{Rx}},D\right)$ is the set of all nonnegative pairs $\left(S^{\left(F\right)},S^{\left(S\right)}\right)$ satisfying*

(15) $$\begin{array}{cc} 2S^{\left(F\right)}+S^{\left(S\right)} & \leq L \end{array}$$

(16) $$\begin{array}{cc} S^{\left(F\right)}+S^{\left(S\right)} & \leq \min\left\{\frac{L}{2}+\mu_{Rx},\;\;L\cdot \frac{2D+1}{2D+2}\right\}. \end{array}$$

**Proof.**  The converse to (16) follows by extending the proof in Reference [12] to the multi-antenna case and by noting that the sum MG of “slow” and “fast” messages cannot be larger than the MG of a scenario with only “slow” messages. The converse to (15) is proved in Appendix A. For the achievability, define the following five MG pairs:

(17a) $$\begin{array}{ccc} (S^{\left(F\right)} & = & \frac{L}{2},\;S^{\left(S\right)}=0), \end{array}$$

(17b) $$\begin{array}{ccc} (S^{\left(F\right)} & = & 0,\;S^{\left(S\right)}=L\cdot \frac{2D+1}{2D+2}), \end{array}$$

(17c) $$\begin{array}{ccc} (S^{\left(F\right)} & = & 0,\;S^{\left(S\right)}=\frac{L}{2}+\mu_{\mathrm{Rx}}), \end{array}$$

(17d) $$\begin{array}{ccc} (S^{\left(F\right)} & = & \frac{L}{2D+2},\;S^{\left(S\right)}=L\cdot \frac{2D}{2D+2}), \end{array}$$

(17e) $$\begin{array}{ccc} (S^{\left(F\right)} & = & \frac{L}{2}-\mu_{\mathrm{Rx}},\;S^{\left(S\right)}=2\mu_{\mathrm{Rx}}). \end{array}$$

In the following Section 3.1.1 we show that when $\mu_{\mathrm{Rx}}\geq \mu_{\max}$, where

(18) $$\mu_{\max}≜L\cdot \frac{D}{2D+2},$$

the MG pairs (17a,b,d) are achievable. When $\mu_{\mathrm{Rx}}<\mu_{\max}$ the MG pairs (17a,c,e) are achievable. The proof of achievability of Theorem 1 then follows from simple time-sharing arguments. □

**Figure 2:**
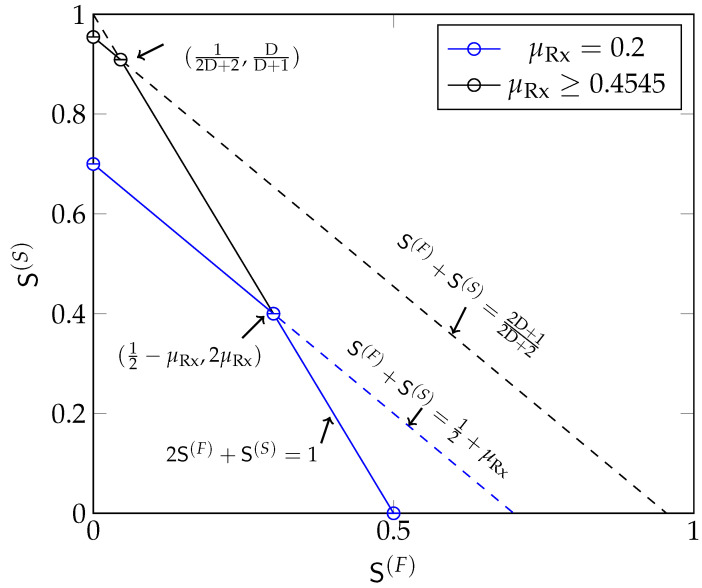
The optimal MG region $S_{\mathrm{Rx}}^{🟉}\left(\mu_{\mathrm{Rx}},D\right)$ for Rx-cooperation only, for different values of $\mu_{\mathrm{Rx}}$ and D=10 and L=1.

Figure 2 depicts the MG region in Theorem 1 for different values of $\mu_{\mathrm{Rx}}$. When there are only “slow” messages, the maximum MG is $\min\{\frac{L}{2}+\mu_{Rx},L\cdot \frac{2D+1}{2D+2}\}$. Notice that in any scheme, we can replace a “fast” message by a “slow” message. By a rate-transfer argument, the maximum sum-MG thus coincides with the maximum “slow” MG. Interestingly, this sum MG remains unchanged whenever the “fast” MG $S^{\left(F\right)}$ is below a certain threshold. Mathematically, this is described by the slope of the boundary of the region being equal to −1 when

(19) $$S^{\left(F\right)}\leq \max\left\{\frac{L}{2}-\mu_{\mathrm{Rx}},\frac{L}{2D+2}\right\}.$$

For

(20) $$S^{\left(F\right)}>\max\left\{\frac{L}{2}-\mu_{\mathrm{Rx}},\frac{L}{2D+2}\right\},$$

the slope is −2. In this latter regime, increasing the MG of “fast” messages by Δ requires decreasing the MG of “slow” messages by 2Δ. There is thus a penalty in sum MG caused by the more stringent delay constraints on “fast” messages.

#### 3.1.1. Schemes Proving Achievability of Theorem 1

We prove achievability of the MG pairs in (17).

**Figure 3:**
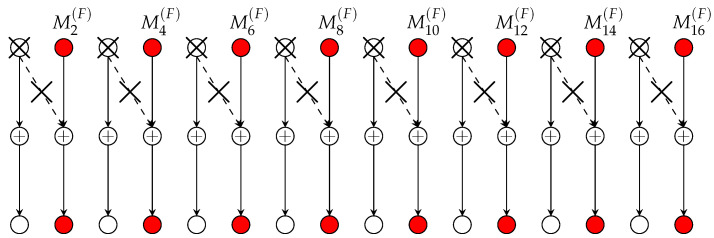
Scheme achieving Multiplexing Gain (MG) pair (17a) where only “fast” messages are transmitted.

1. MG pair in (17a): Periodically silence every second Tx. This splits the network into ⌈K/2⌉ non-interfering point-to-point links. Send a “fast” message over each of these links (see Figure 3), but no “slow” message at all. The described scheme achieves the MG pair in (17a) and requires no cooperation rate.

2. MG pairs in (17b,c): Let the Txs only send “slow” messages but no “fast” messages. Under this coding assumption, the setup at hand is a multi-antenna version of the setup in Reference [12], but specialized to 0 Tx-cooperation rounds and D Rx-cooperation rounds. The multi-antenna extension of the scheme proposed in Reference [12] (Section V) can thus be used to achieve the MG pair in (17b) if $\mu_{\mathrm{Rx}}\geq \mu_{\max}$ and the MG pair in (17c) if $\mu_{\mathrm{Rx}}<\mu_{\max}$.

**Figure 4:**
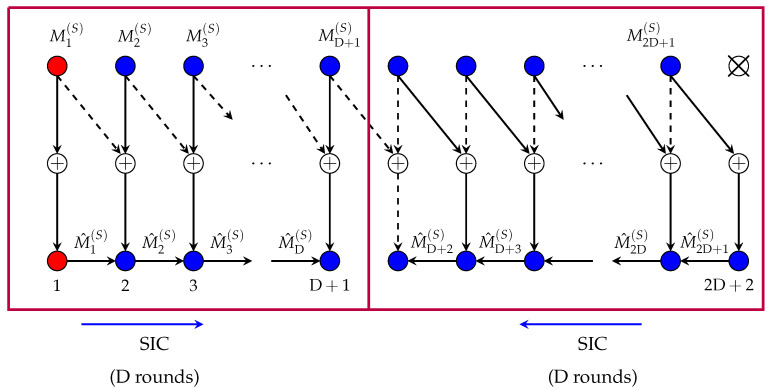
Scheme for Rx-cooperation only.

For reference in the following subsection, we briefly review the scheme in Reference [12] (Section V) when specialized to Rx-cooperation only. For details, see Reference [12]. Consider first the case $\mu_{\mathrm{Rx}}\geq \mu_{\max}$. In this case, the scheme periodically silences every 2D+2nd Tx. This splits the network into smaller subnets, each consisting of 2D+1 active Txs and 2D+2 active Rxs. We describe the communication in the first subnet, see also Figure 4; the others are treated in an analogous way.

Each Tx k∈{1,…,2D+1} in this first subnet encodes its “slow” message $M_{k}^{\left(S\right)}$ using an L-dimensional Gaussian codebook and then sends the resulting codeword using its L Tx-antennas over the channel. Decoding is performed as follows. Rx 1 decodes its desired message using an optimal point-to-point decoding method based on the interference-free channel outputs $Y_{1}^{n}=H_{1,1}X_{1}^{n}+Z_{1}^{n}$. Then it sends its decoded message ${\hat{M}}_{1}^{\left(S\right)}$ over the cooperation link to Rx 2 during the first cooperation round. Rxs 2 to D+1 apply successive interference cancellation (SIC) where they cancel the interference from the preceding Tx with the cooperation message obtained from their left neighbour. After decoding its intended “slow” message, each Rx k∈{2,…,D} sends its decoded message ${\hat{M}}_{k}^{\left(S\right)}$ over the cooperation link to Rx k+1 during cooperation round *k*.

We now describe decoding at Rxs D+2,…,2D+2. Recall that Tx 2D+2 is silenced. Therefore Rx 2D+2 observes the interference-free channel outputs $Y_{2D+2}^{n}=H_{2D+1,2D+2}X_{2D+1}^{n}+Z_{2D+2}^{n}$. Based on these outputs, Rx 2D+2 decodes the “slow” message $M_{2D+1}^{\left(S\right)}$ intended for Rx 2D+1 and transmits the decoded message ${\hat{M}}_{2D+1}^{\left(S\right)}$ to this Rx over the cooperation link in round 1. Rxs D+2 to 2D+1 declare the cooperation message that they receive from their right neighbour as their desired message. They also employ SIC to decode the “slow” message intended for the neighbour to their left. Finally, after this decoding step, each Rx k∈{D+3,…,2D+2} sends the decoded message ${\hat{M}}_{k-1}^{\left(S\right)}$ over the cooperation link to its left neighbour during cooperation round 2D+3−k. Figure 4 illustrates the decodings and conferenced messages.

In the described scheme, 2D+1 Txs send a “slow” message using an L-dimensional Gaussian codebook of power P and all these messages can be decoded based on interference-free outputs. An average “slow” MG of $L\cdot \frac{2D+1}{2D+2}$ is thus achieved in each subnet. Moreover, 2D cooperation messages are sent in each subnet, each of prelog equal to the rate of a “slow” message, i.e., L. The *average* cooperation prelog *per link* is thus $L\cdot \frac{2D}{2(2D+2)}=\mu_{\max}$. If one time-shares 2D+2 different instances of the described scheme with a different subset of silenced users in each of them, the overall scheme achieves the MG pair $\left(S^{\left(F\right)}=0,S^{\left(S\right)}=L\cdot \frac{2D+1}{2D+2}\right)$ with each cooperation link being loaded at average cooperation prelog $\mu_{\max}$.

When $\mu_{\mathrm{Rx}}<\mu_{\max}$, we can time-share the scheme achieving (17b) with a scheme that deactivates every second Tx and sends “slow” messages over the interference-free links. This latter scheme does not require any cooperation. Time-sharing is done according to the available cooperation prelog $\mu_{\mathrm{Rx}}$: the first scheme that uses cooperation prelog $\mu_{\max}$ is used over a fraction $\frac{\mu_{\mathrm{Rx}}}{\mu_{\max}}$ of time and the no-cooperation scheme over the remaining fraction $1-\frac{\mu_{\mathrm{Rx}}}{\mu_{\max}}$ of time. The combined scheme then requires cooperation prelog $\mu_{\mathrm{Rx}}$ and achieves the MG pair in (17c).

3. MG pairs in (17d,e): Reconsider the coding scheme that achieves MG pair (17b) and that is described in the previous subsection and illustrated in Figure 4. A close inspection of the scheme reveals that in each subnet, decoding of the message sent by the left-most Tx does not rely on the conferenced information. This first message of each subnet thus satisfies our decoding requirement for “fast” messages.

We propose to apply the above scheme, but to let the first Tx of every subnet (the red Tx in Figure 4) send a “fast” message and the subsequent 2D Txs of the subnet send “slow” messages. This modified scheme requires the same cooperation prelog $\mu_{\max}$ as before and it achieves the MG pair in (17d).

For setups where $\mu_{\mathrm{Rx}}<\mu_{\max}$, we propose to time-share the scheme achieving (17d) over a fraction $\frac{\mu_{\mathrm{Rx}}}{\mu_{\max}}$ of time with the scheme achieving (17a) over the remaining fraction $1-\frac{\mu_{\mathrm{Rx}}}{\mu_{\max}}$ of time. This time-sharing scheme has cooperation prelog equal to $\mu_{\mathrm{Rx}}$, and thus respects the constraint (10). Moreover, it achieves the MG pair in (17e).

### 3.2. Optimal MG Region and Coding Schemes for Tx-Cooperation Only

**Theorem** **2** (Optimal MG region: Tx-cooperation Only)**.** *For any given $\mu_{\mathrm{Tx}}>0$, the MG region $S_{\mathrm{Tx}}^{🟉}\left(\mu_{\mathrm{Tx}},D\right)$ is the set of all nonnegative pairs $\left(S^{\left(F\right)},S^{\left(S\right)}\right)$ satisfying*

(21) $$\begin{array}{cc} 2S^{\left(F\right)}+S^{\left(S\right)} & \leq L \end{array}$$

(22) $$\begin{array}{cc} S^{\left(F\right)}+S^{\left(S\right)} & \leq \min\left\{\frac{L}{2}+\mu_{Tx},\;\;L\cdot \frac{2D+1}{2D+2}\right\}. \end{array}$$

**Proof.**  The converse to (22) follows by extending the proof in Reference [12] to the multi-antenna case and by noting that the sum MG cannot be larger than the MG of a scenario with only “slow” messages. The converse to (21) is proved in Appendix B. For the achievability, define the following MG pairs:

(23a) $$\begin{array}{ccc} (S^{\left(F\right)} & = & 0,\;S^{\left(S\right)}=L\cdot \frac{2D+1}{2D+2}), \end{array}$$

(23b) $$\begin{array}{ccc} (S^{\left(F\right)} & = & 0,\;S^{\left(S\right)}=\frac{L}{2}+\mu_{\mathrm{Tx}}), \end{array}$$

(23c) $$\begin{array}{ccc} (S^{\left(F\right)} & = & \frac{L}{2D+2},\;S^{\left(S\right)}=L\cdot \frac{2D}{2D+2}), \end{array}$$

(23d) $$\begin{array}{ccc} (S^{\left(F\right)} & = & \frac{L}{2}-\mu_{\mathrm{Tx}},\;S^{\left(S\right)}=2\mu_{\mathrm{Tx}}). \end{array}$$

In the following Section 3.2.1 we show that when $\mu_{\mathrm{Tx}}\geq \mu_{\max}$ the MG pairs (17a) and (23a,c) are achievable and when $\mu_{\mathrm{Tx}}<\mu_{\max}$ the MG pairs (17a) and (23b,d) are achievable. The achievability proof of the theorem then follows by simple time-sharing arguments. □

**Remark** **1.** *Notice the duality between Theorems 1 and 2, which show that cooperation is equally beneficial for only Tx- or only Rx-cooperation. As we will see in Section 4, it is however more beneficial, when Txs* and *Rxs can cooperate.*

#### 3.2.1. Schemes Proving the Achievability of Theorem 2

We prove achievability of the MG pairs in (23). MG pair (17a) is achievable as described in the previous section (no cooperation is required at all).

1. MG pairs in (23a,b): Let the Txs only send “slow” messages but no “fast” messages. Under this coding assumption, the introduced setup corresponds to a multi-antenna version of the setup in Reference [12] but specialized to D Tx-cooperation rounds and 0 Rx-cooperation rounds. Achievability of MG pairs (23a,b) then follows immediately by specializing [12] (Theorem 1) to Tx-cooperation only. In the following we briefly describe the schemes achieving (23a,b). For details see Reference [12].

**Figure 5:**
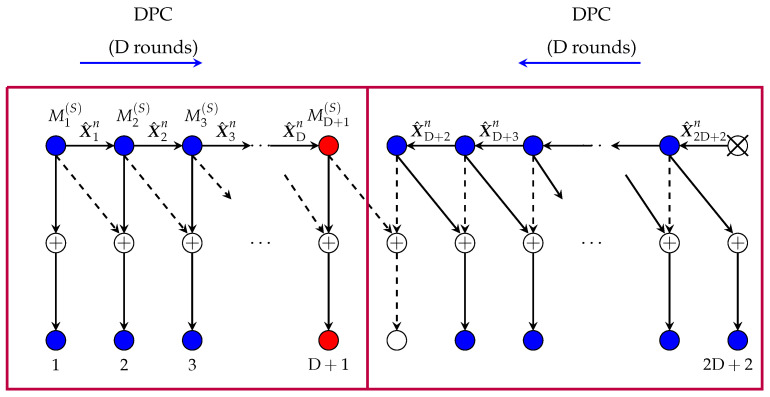
Scheme for Tx-cooperation only.

We silence every 2D+2nd Tx. This splits the network into non-interfering subnets, and in a given subnet we apply the scheme depicted in Figure 5. Specifically, Tx 1 encodes its message using an L-dimensional power-P Gaussian point-to-point codebook, and sends the resulting codeword $X_{1}^{n}$ using its L Tx-antennas over the channel. It also precodes the obtained sequence with the matrix $H_{2,2}^{-1}H_{1,2}$, quantises the precoded sequence $I_{1}^{n}≜H_{2,2}^{-1}H_{1,2}X_{1}^{n}$ with a rate-$\frac{L}{2}\log\left(1+P\right)$ quantiser to obtain a quantisation $\hat{I_{1}^{n}}$ at noise level, and sends the resulting quantisation message as a first-round cooperation message to Tx 2. For each k=2,…,D+1, Tx *k* obtains a round-(k−1) cooperation message from its left neighbour Tx k−1 that describes the quantised version ${\hat{I}}_{k-1}^{n}$ of $I_{k-1}^{n}≜H_{k,k}^{-1}H_{k-1,k}X_{k-1}^{n}$. Based on this message, Tx *k* reconstructs ${\hat{I}}_{k-1}^{n}$, encodes its “slow” message $M_{k}^{\left(S\right)}$ using a power P dirty-paper code (DPC) that mitigates the interference ${\hat{I}}_{k-1}^{n}$, and sends the resulting DPC sequence $X_{k}^{n}$ over the channel. Moreover, it precodes this input sequence with the matrix $H_{k+1,k+1}^{-1}H_{k,k+1}$, quantises the precoded sequence $I_{k}^{n}≜H_{k+1,k+1}^{-1}H_{k,k+1}X_{k}^{n}$ with a rate-L/2log(1+P) quantiser (for a quantisation at noise level) to obtain ${\hat{I}}_{k}^{n}$, and sends the quantisation message as a round-*k* cooperation message over the link to its right neighbour. Tx D+1 produces its inputs in a similar way, that is, using DPC, but sends no cooperation message at all.

Rx 1 decodes $M_{1}^{\left(S\right)}$ based on the interference-free outputs

(24) $$Y_{1}^{n}=H_{1,1}X_{1}^{n}+Z_{1}^{n},$$

using a standard point-to-point decoding rule. Each Rx k∈{2,…,D+1} decodes its desired message $M_{k}^{\left(S\right)}$ based on the premultiplied outputs

(25) $$H_{k,k}^{-1}Y_{k}^{n}=H_{k,k}^{-1}H_{k-1,k}X_{k-1}^{n}+X_{k}^{n}+H_{k,k}^{-1}Z_{k}^{n},$$

using an optimal DPC decoding rule. (Recall that $X_{k}^{n}$ was produced as a DPC sequence that mitigates ${\hat{I}}_{k-1}^{n}$, a quantised version of $I_{k-1}^{n}=H_{k,k}^{-1}H_{k-1,k}X_{k-1}^{n}$). Since quantisation was performed at noise level, each message $M_{1}^{\left(S\right)},\ldots ,M_{D+1}^{\left(S\right)}$ can be sent reliably with MG L.

Each message $M_{k}$, with k∈{D+3…2D+2}, is sent over the path Tx k→Tx k−1→ Rx *k*. We describe the transmissions in more detail, starting with the last Tx in the subnet. Tx 2D+2 does not send any channel inputs, that is, $X_{2D+2}^{n}=0^{n}$. However, it first encodes its “slow” message $M_{2D+2}^{\left(S\right)}$ using an L-dimensional Gaussian point-to-point codebook, precodes the codeword $U_{2D+2}^{n}$ by the matrix $H_{2D+1,2D+2}^{-1}$, and then quantises this precoded codeword $S_{2D+1}^{n}≜H_{2D+1,2D+2}^{-1}U_{2D+2}^{n}$ with a rate-L/2log(1+P) to obtain a quantisation ${\hat{S}}_{2D+1}^{n}$ at noise level. It finally sends the quantisation message describing ${\hat{S}}_{2D+1}^{n}$ as a first-round cooperation message to Tx 2D+1. Tx 2D+1 reconstructs ${\hat{S}}_{2D+1}^{n}$ and sends it over the channel, that is, $X_{2D+1}^{n}={\hat{S}}_{2D+1}^{n}$.

In a similar way, each Tx k∈{2D+1,…,D+2} encodes its own “slow” message $M_{k}^{\left(S\right)}$ by means of DPC of power P that mitigates the interference $H_{k-1,k}^{-1}H_{k,k}X_{k}^{n}$ of the signal sent by Tx *k* itself; precodes the obtained sequence $U_{k}^{n}$ with the matrix $H_{k-1,k}^{-1}H_{k,k}$; quantises the precoded sequence $S_{k-1}^{n}≜H_{k-1,k}^{-1}H_{k,k}U_{k}^{n}$ to obtain a quantisation ${\hat{S}}_{k-1}$ at noise level; and sends the corresponding quantisation message as a (2D+3−k)-round cooperation message over the link to Tx k−1. Tx k−1 then reconstructs ${\hat{S}}_{k-1}^{n}$ and sends it over the channel: $X_{k-1}^{n}={\hat{S}}_{k-1}^{n}$. Rxs D+2,…,2D+1 decode their intended messages using an optimal DPC decoding rule based on the premultiplied outputs

(26) $$H_{k-1,k}^{-1}Y_{k}^{n}=X_{k-1}^{n}+H_{k-1,k}^{-1}H_{k,k}X_{k}^{n}+H_{k-1,k}^{-1}Z_{k}^{n}.$$

Recall that $X_{k-1}^{n}$ is a quantised version (at noise level) of the precoded signal $S_{k-1}^{n}≜H_{k-1,k}^{-1}H_{k,k}U_{k}^{n}$, where $U_{k}^{n}$ is a DPC sequence that mitigates the interference $H_{k-1,k}^{-1}H_{k,k}X_{k}^{n}$. Each of the messages $M_{D+3}^{\left(S\right)},\ldots ,M_{2D+2}^{\left(S\right)}$ can thus be transmitted reliably at full MG L.

In the described scheme, an average “slow” MG of $L\cdot \frac{2D+1}{2D+2}$ is thus achieved in each subnet. Moreover, 2D cooperation messages of prelog L are sent in each subnet, and the *average* cooperation prelog *per link* is $L\cdot \frac{2D}{2(2D+2)}=\mu_{\max}$. If one time-shares 2D+2 different instances of the described scheme with a different subset of silenced users in each of them, the overall scheme achieves the MG pair $\left(S^{\left(F\right)}=0,S^{\left(S\right)}=L\frac{2D+1}{2D+2}\right)$ with each cooperation link being loaded at average cooperation prelog $\mu_{\max}$.

When $\mu_{\mathrm{Tx}}<\mu_{\max}$, we propose to time-share above described scheme over a fraction $\frac{\mu_{\mathrm{Tx}}}{\mu_{\max}}$ of time with a scheme that deactivates every second Tx and sends “slow” messages over the interference-free links (which does not require any cooperation) over the remaining fraction $1-\frac{\mu_{\mathrm{Tx}}}{\mu_{\max}}$ of time. The overall time-sharing scheme achieves the MG pair (23b) and loads each Tx-cooperation link at prelog $\mu_{\mathrm{Tx}}$.

2. MG pairs in (23c,d): A close inspection of the coding scheme described above and depicted in Figure 5 reveals that in each subnet, the message pertaining to the D+1st Tx does not participate in the cooperation, see Figure 5. That means, all conferenced information is independent of this message. The message thus satisfies the constraints imposed on “fast” messages in our scenario. We thus propose to employ above scheme, but where the D+1st Tx in each subnet (the red Tx in Figure 5) sends a “fast” message and the first and the last D Txs in the subnet send “slow” messages. This scheme requires again cooperation prelog $\mu_{\max}$ and achieves the MG pair in (23c).

When $\mu_{\mathrm{Tx}}<\mu_{\max}$, we can time-share this scheme over a fraction $\frac{\mu_{\mathrm{Tx}}}{\mu_{\max}}$ of time with the scheme achieving (17a) over the remaining fraction $1-\frac{\mu_{\mathrm{Tx}}}{\mu_{\max}}$ of time. The time-shared scheme achieves the MG pair (23d) and loads each Tx-cooperation link at orelog $\mu_{\mathrm{Tx}}$.

## 4. Both Tx-and Rx-Cooperation

In this section we consider both Tx- and Rx-cooperation. Recall that the number of Tx- and Rx-cooperation rounds $D_{\mathrm{Tx}}$ and $D_{\mathrm{Rx}}$ is a design parameter over which we can optimize subject to the sum-constraint $D_{\mathrm{Tx}}+D_{\mathrm{Rx}}\leq D$. For simplicity, in this section we assume that the total number of cooperation rounds D is even.

In Section 4.1 we present our inner and outer bounds on the MG region. We also prove that they match in some cases. In the following subsections we then present the coding schemes that allow us to conclude our achievability result.

### 4.1. Results on MG Region

Let the maximum number of total cooperation rounds D be given. For any pair $D_{\mathrm{Rx}}\in \left\{1,\ldots ,D-1\right\}$ and $D_{\mathrm{Tx}}\in \left\{1,\ldots ,D-1\right\}$ summing to less than D, define

(27) $$\begin{array}{ccc} \mu_{\mathrm{Tx},L}\left(D_{\mathrm{Tx}}\right) & ≜ & L\cdot \frac{D_{\mathrm{Tx}}}{2D+2}, \end{array}$$

(28) $$\begin{array}{ccc} \mu_{\mathrm{Rx},L}\left(D_{\mathrm{Rx}}\right) & ≜ & L\cdot \frac{D_{\mathrm{Rx}}}{2D+2}, \end{array}$$

(29) $$\begin{array}{ccc} \mu_{\mathrm{Tx},H}\left(D_{\mathrm{Tx}}\right) & ≜ & L\cdot \frac{\frac{D}{2}+\frac{3}{4}D_{\mathrm{Tx}}-\frac{1}{4}}{2D+2}, \end{array}$$

(30) $$\begin{array}{ccc} \mu_{\mathrm{Rx},H}\left(D_{\mathrm{Rx}}\right) & ≜ & L\cdot \frac{\frac{D}{2}+D_{\mathrm{Rx}}-1}{2D+2}. \end{array}$$

Notice that $\mu_{\mathrm{Tx},L}\left(D_{\mathrm{Tx}}\right)\leq \mu_{\mathrm{Tx},H}\left(D_{\mathrm{Tx}}\right)$ and $\mu_{\mathrm{Rx},L}\left(D_{\mathrm{Rx}}\right)\leq \mu_{\mathrm{Rx},H}\left(D_{\mathrm{Rx}}\right)$.

Also, define the five MG pairs:

(31a) $$\begin{array}{ccc} S_{\mathrm{NoCoop}}^{\left(F\right)} & ≜ & \left(S^{\left(F\right)}=\frac{L}{2},\;S^{\left(S\right)}=0\right), \end{array}$$

(31b) $$\begin{array}{ccc} S_{\mathrm{NoCoop}}^{\left(S\right)} & ≜ & \left(S^{\left(F\right)}=0,\;S^{\left(S\right)}=\frac{L}{2}\right), \end{array}$$

(31c) $$\begin{array}{ccc} S_{\mathrm{Coop}} & ≜ & \left(S^{\left(F\right)}=0,\;S^{\left(S\right)}=L\cdot \frac{2D+1}{2D+2}\right), \end{array}$$

(31d) $$\begin{array}{ccc} S_{\mathrm{Partial}} & ≜ & \left(S^{\left(F\right)}=L\cdot \frac{2}{2D+2},\;S^{\left(S\right)}=L\cdot \frac{2D-1}{2D+2}\right), \end{array}$$

(31e) $$\begin{array}{ccc} S_{\mathrm{Interlaced}} & ≜ & \left(S^{\left(F\right)}=\frac{L}{2},\;S^{\left(S\right)}=L\cdot \frac{D}{2D+2}\right). \end{array}$$

Notice that all these MG pairs do not depend on the number of cooperation rounds $D_{\mathrm{Tx}}$ and $D_{\mathrm{Rx}}$. In what follows, we will be interested in convex combinations of these points and therefore define for each α∈[0,1]:

(32a) $$\begin{array}{ccc} S_{\mathrm{Coop}}\left(\alpha \right) & ≜ & \alpha \cdot S_{\mathrm{Coop}}+\left(1-\alpha \right)\cdot S_{\mathrm{NoCoop}}^{\left(S\right)}, \end{array}$$

(32b) $$\begin{array}{ccc} S_{\mathrm{Partial}}\left(\alpha \right) & ≜ & \alpha \cdot S_{\mathrm{Partial}}+\left(1-\alpha \right)\cdot S_{\mathrm{NoCoop}}^{\left(F\right)}, \end{array}$$

(32c) $$\begin{array}{ccc} S_{\mathrm{Interlaced}}\left(\alpha \right) & ≜ & \alpha \cdot S_{\mathrm{Interlaced}}+\left(1-\alpha \right)\cdot S_{\mathrm{NoCoop}}^{\left(F\right)}, \end{array}$$

(32d) $$\begin{array}{ccc} S_{\text{Partial-Inter}}\left(\alpha \right) & ≜ & \alpha \cdot S_{\mathrm{Interlaced}}+\left(1-\alpha \right)\cdot S_{\mathrm{Partial}}. \end{array}$$

Notice that $S_{\mathrm{Coop}}\left(1\right)=S_{\mathrm{Coop}}$ and $S_{\mathrm{Partial}}\left(1\right)=S_{\mathrm{Partial}}$ and $S_{\mathrm{Interlaced}}\left(1\right)=S_{\mathrm{Interlaced}}$. Moreover, $S_{\text{Partial-Inter}}\left(1\right)=S_{\mathrm{Interlaced}}$ and $S_{\text{Partial-Inter}}\left(0\right)=S_{\mathrm{Partial}}$.

**Theorem** **3** (Achievable MG Region: Tx- and Rx-cooperation)**.** *For any choice of odd-valued integers $D_{Tx},D_{Rx}\in \left\{1,3,5,\ldots ,D-1\right\}$ summing to D, the optimal MG region $S^{🟉}\left(\mu_{\mathrm{Tx}},\mu_{\mathrm{Rx}},D\right)$ contains some of the following regions, depending on the available cooperation prelogs $\mu_{\mathrm{Tx}}$ and $\mu_{\mathrm{Rx}}$.*

- *If $\mu_{\mathrm{Tx}}\geq \mu_{Tx,H}\left(D_{Tx}\right)$ and $\mu_{\mathrm{Rx}}\geq \mu_{Rx,H}\left(D_{Rx}\right)$, the optimal MG region $S^{🟉}\left(\mu_{\mathrm{Tx}},\mu_{\mathrm{Rx}},D\right)$ contains the trapezoidal region*
  (33) $$\mathrm{conv}\;\mathrm{hull}\left(\left(0,0\right),\;S_{\mathrm{NoCoop}}^{\left(F\right)},\;S_{\mathrm{Coop}},\;S_{\mathrm{Interlaced}}\right).$$
- *If $\mu_{\mathrm{Tx}}\geq \mu_{Tx,L}\left(D_{Tx}\right)$ and $\mu_{\mathrm{Rx}}\geq \mu_{Rx,L}\left(D_{Rx}\right)$, the optimal MG region $S^{🟉}\left(\mu_{\mathrm{Tx}},\mu_{\mathrm{Rx}},D\right)$ contains the pentagon*
  (34) $$\mathrm{conv}\;\mathrm{hull}\left(\left(0,0\right),\;S_{\mathrm{NoCoop}}^{\left(F\right)},\;S_{\mathrm{Coop}},\;S_{\text{Partial-Inter}}\left(\alpha_{1}^{🟉}\right),\;S_{\mathrm{Interlaced}}\left(\beta_{1}^{🟉}\right)\right),$$
  *where*
  (35) $$\alpha_{1}^{🟉}≜\min\left\{\frac{\mu_{\mathrm{Tx}}-\mu_{Tx,L}\left(D_{Tx}\right)}{\mu_{Tx,H}\left(D_{Tx}\right)-\mu_{Tx,L}\left(D_{Tx}\right)},\frac{\mu_{\mathrm{Rx}}-\mu_{Rx,L}\left(D_{Rx}\right)}{\mu_{Rx,H}\left(D_{Rx}\right)-\mu_{Rx,L}\left(D_{Rx}\right)},1\right\}$$
  *and*
  (36) $$\beta_{1}^{🟉}≜\min\left\{\frac{\mu_{\mathrm{Tx}}}{\mu_{Tx,H}\left(D_{Tx}\right)},\frac{\mu_{\mathrm{Rx}}}{\mu_{Rx,H}\left(D_{Rx}\right)},1\right\}.$$
- *For $\mu_{\mathrm{Tx}}\leq \mu_{Tx,L}\left(D_{Tx}\right)$ or $\mu_{\mathrm{Rx}}\leq \mu_{Rx,L}\left(D_{Rx}\right)$, the optimal MG region $S^{🟉}\left(\mu_{\mathrm{Tx}},\mu_{\mathrm{Rx}},D\right)$ contains the region*
  (37) $$\mathrm{conv}\;\mathrm{hull}\left(\left(0,0\right),\;S_{\mathrm{Coop}}\left(\alpha_{2}^{🟉}\right),\;S_{\mathrm{Partial}}\left(\alpha_{2}^{🟉}\right),\;S_{\mathrm{Interlaced}}\left(\beta_{2}^{🟉}\right)\right).$$
  *where*
  (38) $$\alpha_{2}^{🟉}≜\min\left\{\frac{\mu_{\mathrm{Tx}}}{\mu_{Tx,L}\left(D_{Tx}\right)},\frac{\mu_{\mathrm{Rx}}}{\mu_{Rx,L}\left(D_{Rx}\right)}\right\}$$
  *and*
  (39) $$\beta_{2}^{🟉}=\min\left\{\frac{\mu_{\mathrm{Tx}}}{\mu_{Tx,H}\left(D_{Tx}\right)},\frac{\mu_{\mathrm{Rx}}}{\mu_{Rx,H}\left(D_{Rx}\right)}\right\}.$$

**Figure 6:**
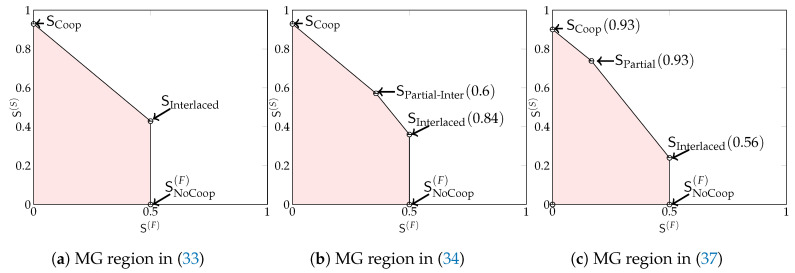
Examples of the three MG regions in (33), (34) and (37). Specifically, we used $D_{\mathrm{Tx}}=3$, $D_{\mathrm{Rx}}=3$, and $\mu_{\mathrm{Tx}}\in \left\{0.4,0.3,0.2\right\}$ and $\mu_{\mathrm{Rx}}\in \left\{0.4,0.3,0.2\right\}$.

In Figure 6 we schematically illustrate above MG regions (33), (34) and (37). We see that for large cooperation prelogs our MG region is the trapezoid in Figure 6a. For smaller cooperation prelogs the MG region turns into a pentagon, see Figure 6b, because MG pair $S_{\mathrm{Interlaced}}$ is not included anymore. Finally, for even smaller cooperation prelogs even the MG pair $S_{\mathrm{Coop}}$ is not included anymore, but needs to be replaced by $S_{\mathrm{Coop}}\left(0.93\right)$. Similarly, $S_{\text{Partial-Inter}}\left(0.6\right)$ needs to be replaced by $S_{\mathrm{Partial}}\left(0.93\right)$.

The achievable MG region described in the theorem can also be written as a union over the choice of the Tx- and Rx-cooperation rounds $D_{\mathrm{Tx}}$ and $D_{\mathrm{Rx}}$ summing to no more than D. Notice however, that one cannot take the convex hull of this union because the way we defined the problem setup the choice of $D_{\mathrm{Tx}}$ and $D_{\mathrm{Rx}}$ needs to be fixed in advance and time-sharing between different choices is not possible.

**Proof** **of** **Theorem** **3**. In the following Section 4.2, Section 4.3 and Section 4.4 we show how to achieve the MG pairs in (31c–e) with sufficiently large cooperation prelogs $\mu_{\mathrm{Tx}}$ and $\mu_{\mathrm{Rx}}$. In particular, to achieve (31c,d), cooperation prelogs $\mu_{\mathrm{Tx}}\geq \mu_{\mathrm{Tx},L}\left(D_{\mathrm{Tx}}\right)$ and $\mu_{\mathrm{Rx}}\geq \mu_{\mathrm{Rx},L}\left(D_{\mathrm{Rx}}\right)$ are required. To achieve (31e) cooperation prelogs $\mu_{\mathrm{Tx}}\geq \mu_{\mathrm{Tx},H}\left(D_{\mathrm{Tx}}\right)$ and $\mu_{\mathrm{Rx}}\geq \mu_{\mathrm{Rx},H}\left(D_{\mathrm{Rx}}\right)$ are required. MG pairs (31a,b) can be achieved without any Tx- or Rx-cooperation by simply silencing every second transmitter and sending either only “fast” or only “slow” messages over the remaining K/2 isolated point-to-point links.

The proof of the theorem follows then by simple time-sharing arguments. In particular, for any α∈[0,1] the MG pair $S_{\mathrm{Coop}}\left(\alpha \right)$ can be achieved by time-sharing the scheme achieving $S_{\mathrm{Coop}}$ over a fraction α of the time with the scheme achieving $S_{\mathrm{NoCoop}}^{\left(S\right)}$ over the remaining fraction of time. Such a time-sharing scheme requires cooperation prelogs of $\mu_{\mathrm{Tx}}\geq \alpha \mu_{\mathrm{Tx},L}$ and $\mu_{\mathrm{Rx}}\geq \alpha \mu_{\mathrm{Rx},L}$. The MG pairs $S_{\mathrm{Partial}}\left(\alpha \right)$ and $S_{\mathrm{Interlaced}}\left(\alpha \right)$ are achieved by time-sharing the scheme achieving $S_{\mathrm{Partial}}$ or the scheme achieving $S_{\mathrm{Interlaced}}$ over a fraction α of the time with the scheme achieving $S_{\mathrm{NoCoop}}^{\left(F\right)}$ over the remaining fraction of time. The time-sharing scheme leading to $S_{\mathrm{Partial}}\left(\alpha \right)$ requires cooperation prelogs $\mu_{\mathrm{Tx}}\geq \alpha \mu_{\mathrm{Tx},L}$ and $\mu_{\mathrm{Rx}}\geq \alpha \mu_{\mathrm{Rx},L}$ and the time-sharing scheme leading to $S_{\mathrm{Interlaced}}\left(\alpha \right)$ requires $\mu_{\mathrm{Tx}}\geq \alpha \mu_{\mathrm{Tx},H}$ and $\mu_{\mathrm{Rx}}\geq \alpha \mu_{\mathrm{Rx},H}$. The MG pair $S_{\text{Partial-Inter}}\left(\alpha \right)$ is achieved by time-sharing the scheme achieving $S_{\mathrm{Interlaced}}$ over a fraction α of the time with the scheme achieving $S_{\mathrm{Partial}}$ over the remaining fraction of time. This time-sharing schme requires cooperation prelogs $\mu_{\mathrm{Tx}}\geq \alpha \mu_{\mathrm{Tx},H}+\left(1-\alpha \right)\mu_{\mathrm{Tx},L}$ and $\mu_{\mathrm{Rx}}\geq \alpha \mu_{\mathrm{Rx},H}+\left(1-\alpha \right)\mu_{\mathrm{Rx},L}$.

Notice that for all of above time-sharing arguments, it is important that the MG pairs $S_{\mathrm{NoCoop}}^{\left(F\right)}$, $S_{\mathrm{NoCoop}}^{\left(S\right)}$, $S_{\mathrm{Coop}}$, $S_{\mathrm{Partial}}$, and $S_{\mathrm{Interlaced}}$ can be achieved using the same values of $D_{\mathrm{Tx}}$ and $D_{\mathrm{Rx}}$. As will become clear in the following sections, these MG pairs can be achieved using any values $D_{\mathrm{Tx}},D_{\mathrm{Rx}}\in \left\{1,3,5,\ldots D-1\right\}$ summing to D. The required cooperation prelogs however depend on the specific choices of $D_{\mathrm{Tx}}$ and $D_{\mathrm{Rx}}$. This explains why the allowed time-sharing coefficients α depend on the number of cooperation rounds $D_{\mathrm{Tx}}$ and $D_{\mathrm{Rx}}$. □

**Remark** **2.** *If in Theorem 3 we allow the parameters $D_{Tx},D_{Rx}$ to take on any values in {1,2,…,D−1} summing to D and we remove the MG points $S_{\mathrm{Interlaced}}$, $S_{\mathrm{Interlaced}}\left(\beta_{1}^{🟉}\right)$, $S_{\mathrm{Interlaced}}\left(\beta_{2}^{🟉}\right)$, and $S_{\text{Partial-Inter}}\left(\alpha_{1}^{🟉}\right)$, we obtain a different achievable region, which can be larger for certain system parameters.*

*To see that this modified region is also achievable, notice that our schemes achieving $S_{\mathrm{Coop}}$ and $S_{\mathrm{Partial}}$ described in Section 4.2 and Section 4.3 can be run with any number of Tx- and Rx- cooperation rounds $D_{Tx}$ and $D_{Rx}$, irrespective of whether they are odd or even. Their performance remains unchanged. In contrast, the scheme achieving $S_{\mathrm{Interlaced}}$ that we present in Section 4.4 requires that both $D_{Tx}$ and $D_{Rx}$ are both odd.*

**Figure 7:**
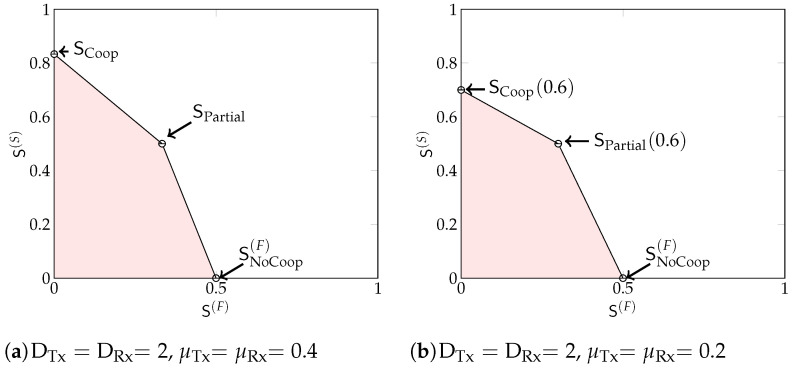
Examples of the MG regions discussed in Remark 2 for even values of $D_{\mathrm{Tx}}$ and $D_{\mathrm{Rx}}$.

*In Figure 7 we schematically illustrate the MG regions that are achieved for $D_{Tx}$ or $D_{Rx}$ even. Specifically, Figure 7a shows the MG region for large cooperation prelogs and Figure 7b for small cooperation prelogs.*

We also have the following converse result.

**Proposition** **1** (Outer Bound on Optimal MG Region: Both Tx- and Rx-cooperation)**.** *Any MG pair $\left(S^{\left(F\right)},S^{\left(S\right)}\right)$ in $S^{🟉}\left(\mu_{\mathrm{Tx}},\mu_{\mathrm{Rx}},D\right)$ satisfies*

(40a) $$\begin{array}{cc} S^{\left(F\right)} & \leq \frac{L}{2}, \end{array}$$

(40b) $$\begin{array}{cc} S^{\left(F\right)}+S^{\left(S\right)} & \leq \min\left\{\frac{L}{2}+\mu_{\mathrm{Tx}}+\mu_{\mathrm{Rx}},\;L\cdot \frac{2D+1}{2D+2}\right\}. \end{array}$$

**Proof.**  Follows from the converse result in Reference [12] and by a rate-transfer argument from “fast” to “slow” messages. □

**Figure 8:**
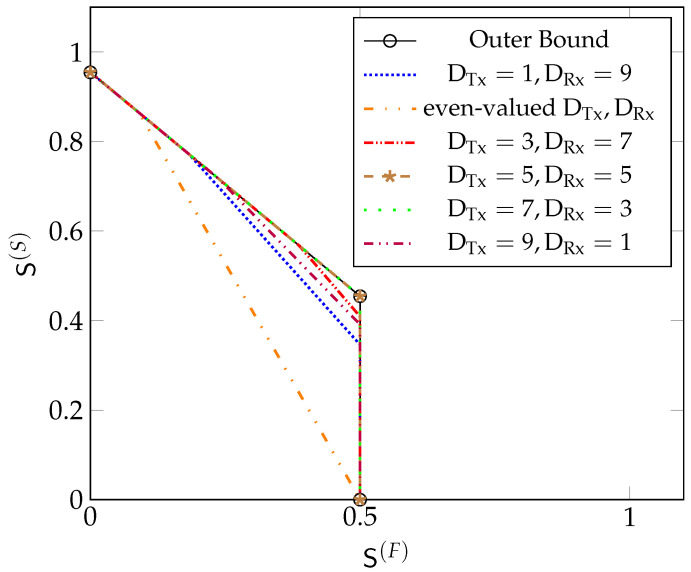
Bounds on $S^{🟉}\left(\mu_{\mathrm{Tx}},\mu_{\mathrm{Rx}},D\right)$ for $\mu_{\mathrm{Tx}}=0.45$, $\mu_{\mathrm{Rx}}=0.45$, D=10 and L=1 with both Tx- and Rx-cooperation.

Figure 8 depicts our inner and outer bounds (Theorem 3, Remark 2, and Proposition 1) on the optimal MG region with $\mu_{\mathrm{Tx}}=\mu_{\mathrm{Rx}}=0.45$ and D=10 for different values of $D_{\mathrm{Tx}}$ and $D_{\mathrm{Rx}}$. For $D_{\mathrm{Rx}}=D/2=5$ and $D_{\mathrm{Tx}}=D/2=5$,

(41) $$\begin{array}{c} \mu_{\mathrm{Tx}}\geq \mu_{\mathrm{Tx},H}\;\mathrm{and}\;\mu_{\mathrm{Rx}}\geq \mu_{\mathrm{Rx},H}, \end{array}$$

and the inner bound is given by the trapezoidal region defined in (33). It coincides with the outer bound, and thus establishes the exact MG region. Notice that in this case, the MG region is solely constrained by the fact that the MG of “fast” messages cannot exceed $\frac{L}{2}$ and that the sum MG of all messages cannot exceed $L\cdot \frac{2D_{\mathrm{Rx}}+2D_{\mathrm{Tx}}+1}{2D_{\mathrm{Rx}}+2D_{\mathrm{Tx}}+2}$. Imposing a stringent constraint on the decoding delay of the “fast” messages in this case never penalises the sum-MG of the system. Our inner bounds obtained for odd-valued cooperation-rounds $\left(D_{\mathrm{Tx}},D_{\mathrm{Rx}}\right)\in \left\{\left(1,9\right),\left(3,7\right),\left(7,3\right),\left(9,1\right)\right\}$ coincide with the outer bound only if $S^{\left(F\right)}\leq L\cdot \frac{2\left(1-\alpha_{1}^{🟉}\right)+\alpha_{1}^{🟉}\cdot \left(D+1\right)}{2D+2}$, where $\alpha_{1}^{🟉}$ depends on the choice of $\left(D_{\mathrm{Tx}},D_{\mathrm{Rx}}\right)$ and is defined in (35). The inner bounds for even-valued cooperation-rounds $\left(D_{\mathrm{Tx}},D_{\mathrm{Rx}}\right)\in \left\{\left(2,8\right),\left(4,6\right),\left(6,4\right),\left(8,2\right)\right\}$ all coincide and attain the outer bound only if $S^{\left(F\right)}\leq L\cdot \frac{2}{2D+2}$.

**Figure 9:**
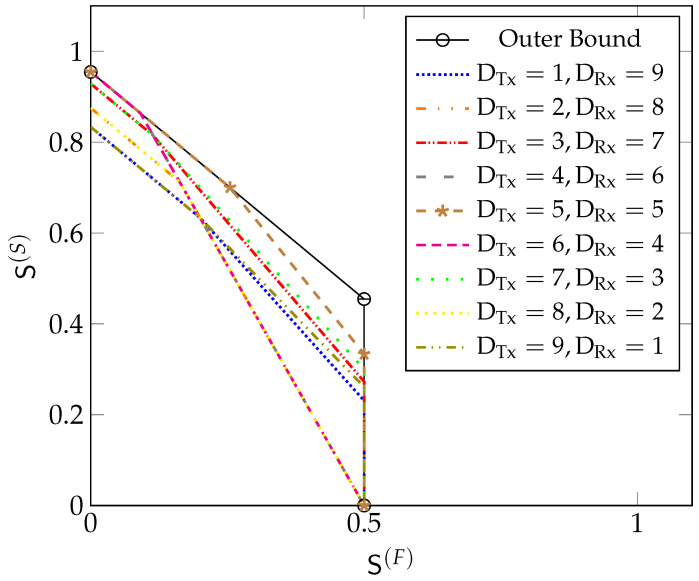
Bounds on $S^{🟉}\left(\mu_{\mathrm{Tx}},\mu_{\mathrm{Rx}},D\right)$ for $\mu_{\mathrm{Tx}}=0.3$, $\mu_{\mathrm{Rx}}=0.3$, D=10 and L =1 with both Tx- and Rx-cooperation.

Figure 9 depicts our inner and outer bounds on the optimal MG region for the same D=10 but smaller values of $\mu_{\mathrm{Tx}}=\mu_{\mathrm{Rx}}=0.3$. In Figure 9, we see that by decreasing $\mu_{\mathrm{Tx}}$ and $\mu_{\mathrm{Rx}}$ from 0.45 to 0.3, our inner and outer bounds do not coincide for all values of $S^{\left(F\right)}$. Our inner bound for $D_{\mathrm{Rx}}=5$ and $D_{\mathrm{Tx}}=5$ contains all other inner bounds and it matches the outer bound in the regime $S^{\left(F\right)}\leq L\cdot \frac{2\left(1-\alpha_{1}^{🟉}\right)+\alpha_{1}^{🟉}\cdot \left(D+1\right)}{2D+2}$, where for the definition of $\alpha_{1}^{🟉}$ in (35) one should set $D_{\mathrm{Tx}}=D_{\mathrm{Rx}}=5$.

**Figure 10:**
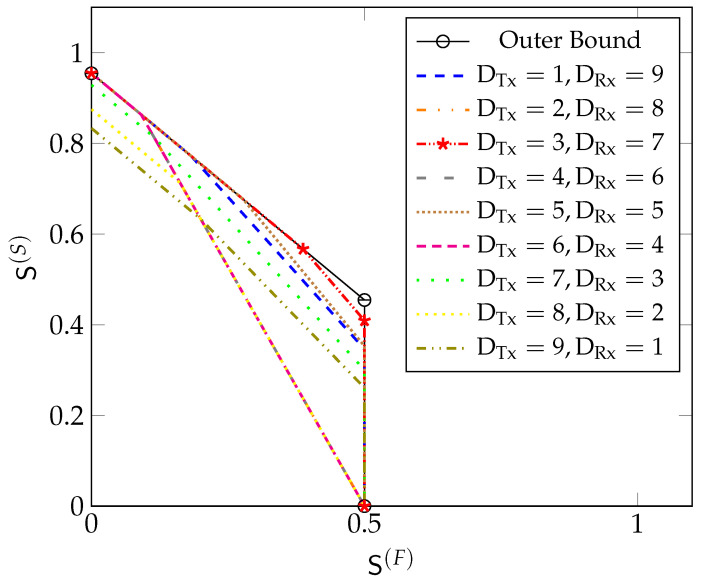
Bounds on $S^{🟉}\left(\mu_{\mathrm{Tx}},\mu_{\mathrm{Rx}},D\right)$ for $\mu_{\mathrm{Tx}}=0.3$, $\mu_{\mathrm{Rx}}=0.45$, D=10 and L=1 with both Tx- and Rx-cooperation.

Figure 10 depicts our inner and outer bounds on the optimal MG region for the same D=10 but when $\mu_{\mathrm{Tx}}=0.3$ is smaller than $\mu_{\mathrm{Rx}}=0.45$. Here, the inner bound obtained for $\left(D_{\mathrm{Tx}}=3,D_{\mathrm{Rx}}=7\right)$ includes all other inner bounds and it matches the outer bound in the regime $S^{\left(F\right)}\leq L\cdot \frac{2\left(1-\alpha_{1}^{🟉}\right)+\alpha_{1}^{🟉}\cdot \left(D+1\right)}{2D+2}$, where $\alpha_{1}^{🟉}$ is defined in (35) with $D_{\mathrm{Tx}}=3$ and $D_{\mathrm{Rx}}=7$.

The following corollaries generalise these observations.

**Corollary** **1.** *If there exist integers $D_{Tx},D_{Rx}\in \left\{1,3,5,\ldots ,D-1\right\}$ summing to D such that the two constraints*

(42a) $$\begin{array}{ccc} \mu_{\mathrm{Tx}} & \geq & \mu_{\mathrm{Tx},H}\left(D_{Tx}\right) \end{array}$$

(42b) $$\begin{array}{ccc} \mu_{\mathrm{Rx}} & \geq & \mu_{\mathrm{Rx},H}\left(D_{Rx}\right) \end{array}$$

*are simultaneously satisfied, then the optimal MG region $S^{🟉}\left(\mu_{\mathrm{Tx}},\mu_{\mathrm{Rx}},D\right)$ coincides with the trapezoidal region in *(33)*. That means, $S^{🟉}\left(\mu_{\mathrm{Tx}},\mu_{\mathrm{Rx}},D\right)$ is the set of all nonnegative pairs $\left(S^{\left(F\right)},S^{\left(S\right)}\right)$ satisfying*

(43) $$\begin{array}{cc} S^{\left(F\right)} & \leq \frac{L}{2}, \end{array}$$

(44) $$\begin{array}{cc} S^{\left(F\right)}+S^{\left(S\right)} & \leq L\cdot \frac{2D+1}{2D+2}. \end{array}$$

**Proof.**  Follows directly from the achievability result in Theorem 3, see (33), and the converse result in Proposition 1. For the converse result notice in particular that under constraints (42) the sum $\mu_{\mathrm{Tx}}+\mu_{\mathrm{Rx}}$ exceeds $L\cdot \frac{D}{2D+2}$. □

**Remark** **3.** *Under conditions *(42)* there is no penalty in sum-MG due to the stringent decoding constraint on “fast” messages. These “fast” messages can be submitted at maximum MG without decreasing the overall performance of the system.*

The following corollaries present partial characterizations of the optimal MG region $S^{🟉}\left(\mu_{\mathrm{Tx}},\mu_{\mathrm{Rx}},D\right)$ for $S^{\left(F\right)}$ below a certain threshold.

**Corollary** **2.** *If a pair of integers $D_{Tx},D_{Rx}\in \left\{1,3,\ldots ,D-1\right\}$ summing to D satisfies*

(45a) $$\begin{array}{ccc} \mu_{\mathrm{Tx}} & \geq & \mu_{\mathrm{Tx},L}\left(D_{Tx}\right), \end{array}$$

(45b) $$\begin{array}{ccc} \mu_{\mathrm{Rx}} & \geq & \mu_{\mathrm{Rx},L}\left(D_{Rx}\right), \end{array}$$

*then the optimal MG region $S^{🟉}\left(\mu_{\mathrm{Tx}},\mu_{\mathrm{Rx}},D\right)$ contains the MG pair $\left(S^{\left(F\right)},S^{\left(S\right)}\right)$ with*

(46) $$S^{\left(F\right)}\leq \frac{L}{2}\left(1-\frac{D-1}{D+1}\left(1-\alpha_{1}^{🟉}\right)\right)$$

*(where $\alpha_{1}^{🟉}$ is defined in *(35*) and depends on the choice of $D_{Tx},D_{Rx}$ and on $\mu_{Tx},\mu_{Rx}$) if, and only if,*

(47) $$S^{\left(F\right)}+S^{\left(S\right)}\leq L\cdot \frac{2D+1}{2D+2}.$$

*Similarly, if a pair of integers $D_{Tx},D_{Rx}\in \left\{2,4,\ldots ,D-2\right\}$ summing to D satisfies (45), then the optimal MG region $S^{🟉}\left(\mu_{\mathrm{Tx}},\mu_{\mathrm{Rx}},D\right)$ contains the MG pair $\left(S^{\left(F\right)},S^{\left(S\right)}\right)$ with*

(48) $$S^{\left(F\right)}\leq \frac{L}{2}\cdot \frac{2}{D+1}$$

*if, and only if,*

(49) $$S^{\left(F\right)}+S^{\left(S\right)}\leq L\cdot \frac{2D+1}{2D+2}.$$

Noting the fundamental bound $S^{\left(F\right)}\leq \frac{L}{2}$, one observes that when there is a odd-valued pair $D_{\mathrm{Tx}},D_{\mathrm{Rx}}\in \left\{1,3,\ldots ,D-1\right\}$ such that (45) holds and $\alpha_{1}^{🟉}=1$, then the first part of Corollary 2 recovers Corollary 1 and determines the entire optimal MG region $S^{🟉}\left(\mu_{\mathrm{Tx}},\mu_{\mathrm{Rx}},D\right)$.

**Proof.**  Achievability of (47) follows from Theorem 3, see (34), because the two components of $S_{\text{Inter-Partial}}\left(\alpha_{1}^{🟉}\right)=\left(S^{\left(S\right)},S^{\left(F\right)}\right)$ satisfy:

(50) $$\begin{array}{c} S^{\left(F\right)}=\alpha_{1}^{🟉}\frac{L}{2}+\left(1-\alpha_{1}^{🟉}\right)\frac{L}{2}\frac{2}{D+1}=\frac{L}{2}\left(1-\frac{D-1}{D+1}\left(1-\alpha_{1}^{🟉}\right)\right) \end{array}$$

and

(51) $$\begin{array}{c} S^{\left(S\right)}+S^{\left(F\right)}=L\cdot \frac{2D+1}{2D+2}. \end{array}$$

Achievability of (49) can be proved in a similar way from Remark 2. The converse to both results follows from Proposition 1 because constraint (47) implies that the sum $\mu_{\mathrm{Tx}}+\mu_{\mathrm{Rx}}$ exceeds $L\cdot \frac{D}{2D+2}$. □

**Corollary** **3.** *If*

(52) $$\begin{array}{ccc} \mu_{\mathrm{Rx}}+\mu_{\mathrm{Tx}} & < & L\cdot \frac{D}{2D+2} \end{array}$$

*and if a pair $D_{Tx},D_{Rx}\in \left\{1,2,3,\ldots ,D-1\right\}$ (both odd and even values are allowed) summing to D satisfies*

(53) $$\begin{array}{ccc} \frac{\mu_{\mathrm{Tx}}}{D_{Tx}} & = & \frac{\mu_{\mathrm{Rx}}}{D_{Rx}}, \end{array}$$

*then the optimal MG region $S^{🟉}\left(\mu_{\mathrm{Tx}},\mu_{\mathrm{Rx}},D\right)$ contains the MG pair $\left(S^{\left(F\right)},S^{\left(S\right)}\right)$ with*

(54) $$S^{\left(F\right)}\leq \frac{L}{2}\left(1-\mu_{Tx}\frac{2(D-1)}{D_{Tx}}\right)$$

*if, and only if,*

(55) $$S^{\left(F\right)}+S^{\left(S\right)}\leq \frac{L}{2}+\mu_{\mathrm{Tx}}+\mu_{\mathrm{Rx}}.$$

**Proof.**  The result (55) follows from the converse result in Proposition 1 and the achievability results in Theorem 3, see (37), and Remark 2. More specifically, to prove achievability let $D_{\mathrm{Tx}}$ and $D_{\mathrm{Rx}}$ be such that Condition (53) is satisfied. Then,

(56) $$\frac{\mu_{\mathrm{Tx},L}\left(D_{\mathrm{Tx}}\right)}{\mu_{\mathrm{Tx}}}=\frac{\mu_{\mathrm{Rx},L}\left(D_{\mathrm{Rx}}\right)}{\mu_{\mathrm{Rx}}}$$

and Condition (52) implies that both inequalities

(57) $$\begin{array}{c} \mu_{\mathrm{Tx}}<\mu_{\mathrm{Tx},L}\;\mathrm{and}\;\mu_{\mathrm{Rx}}<\mu_{\mathrm{Rx},L} \end{array}$$

are satisfied. Moreover, $\alpha_{2}^{🟉}$ as defined in (38) satisfies

(58) $$\alpha_{2}^{🟉}=\frac{\mu_{\mathrm{Tx}}}{\mu_{\mathrm{Tx},L}}=\mu_{\mathrm{Tx}}\frac{2D+2}{L\cdot D_{\mathrm{Tx}}}=\frac{\mu_{\mathrm{Rx}}}{\mu_{\mathrm{Rx},L}}=\mu_{\mathrm{Rx}}\frac{2D+2}{L\cdot D_{\mathrm{Rx}}}.$$

Notice next that for the two MG pairs $S_{\mathrm{Coop}}\left(\alpha_{2}^{🟉}\right)$ and $S_{\mathrm{Partial}}\left(\alpha_{2}^{🟉}\right)$, which are achievable by either Theorem 3 or Remark 2, the sum of the two components satisfies

(59) $$\begin{array}{c} S^{\left(F\right)}+S^{\left(S\right)}=\alpha_{2}^{🟉}\cdot \frac{L}{2}\cdot \frac{2D+1}{D+1}+\left(1-\alpha_{2}^{🟉}\right)\frac{L}{2}=\frac{L}{2}+\frac{\mu_{\mathrm{Tx}}}{D_{\mathrm{Tx}}}D=\frac{L}{2}+\mu_{\mathrm{Tx}}+\mu_{\mathrm{Rx}}, \end{array}$$

where in the last equation we used (53). Moreover, the “fast” MG $S^{\left(F\right)}$ in $S_{\mathrm{Coop}}\left(\alpha_{2}^{🟉}\right)$ equals 0, whereas in $S_{\mathrm{Partial}}\left(\alpha_{2}^{🟉}\right)$ it equals

(60) $$S^{\left(F\right)}=\alpha_{2}^{🟉}\cdot \frac{L}{2}\cdot \frac{2}{D+1}+\left(1-\alpha_{2}^{🟉}\right)\frac{L}{2}=\left(1-\mu_{\mathrm{Tx}}\frac{2(D-1)}{D_{\mathrm{Tx}}}\right)=\left(1-\mu_{\mathrm{Rx}}\frac{2(D-1)}{D_{\mathrm{Rx}}}\right).$$

Since one can always choose to transmit at smaller MGs and because the convex hull of all achievable MG pairs is also achievable, this concludes the proof of achievability. □

**Remark** **4.** *For both corollaries, in the regimes where we could characterize the optimal MG region, i.e., for “fast” MGs below a certain threshold, the sum-MG is at its maximum. We can thus conclude that for sufficiently small $S^{\left(F\right)}$ the sum-MG is not decreased due to the stringent constraint on the “fast” messages.*

In the following subsections we present the coding schemes achieving the MG regions in Theorem 3 and Remark 2.

### 4.2. Scheme Achieving (31c)

Let each Tx only send “slow” messages but no “fast” messages. Under this coding assumption, our setup is a multi-antenna version of the setup in [12]. Achievability of (31c) then follows immediately by the multi-antenna version of [12] (Theorem 1). We redescribe the coding schemes achieving (31c) for completeness and reference in the next subsection.

**Figure 11:**
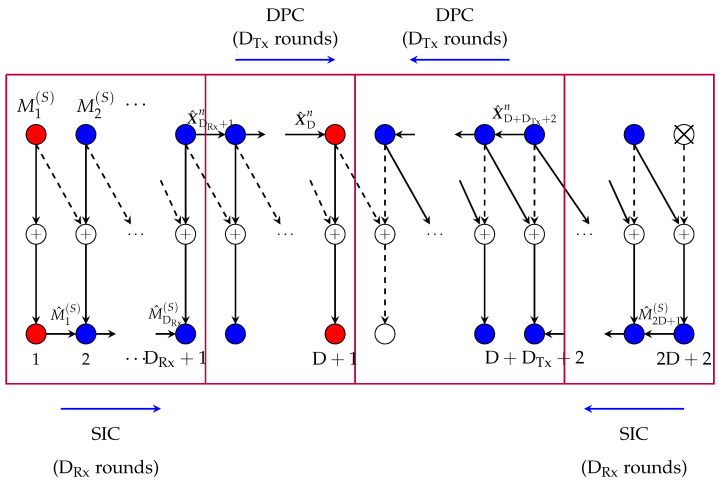
Scheme with Rx- and Tx-cooperation.

We silence every 2D+2nd Tx, which splits the network into smaller subnets. In each subnet, we combine the SIC idea explained for the setup with only Rx-cooperation (see Section 3.1.1) with the DPC coding idea that was explained for the setup with only Tx-cooperation (see Section 3.2.1). The scheme for the first subnet is illustrated in Figure 11 and will be explained in the following. Communication in the other subnets is similar.

The Tx/Rx pairs of the first subnet are assigned to four groups, depending on their mode of operation. Notice that the Tx/Rx pair $D_{\mathrm{Rx}}+2D_{\mathrm{Tx}}+2$ is assigned to both groups $G_{3}$ and $G_{4}$, whereas all other Tx/Rx pairs are assigned to only one group. The reason is that message $M_{D_{\mathrm{Rx}}+2D_{\mathrm{Tx}}+2}^{\left(S\right)}$ is split into two parts $\left(M_{D_{\mathrm{Rx}}+2D_{\mathrm{Tx}}+2}^{\left(S,3\right)},M_{D_{\mathrm{Rx}}+2D_{\mathrm{Tx}}+2}^{\left(S,4\right)}\right)$ of equal rates, and part $M_{D_{\mathrm{Rx}}+2D_{\mathrm{Tx}}+2}^{\left(S,3\right)}$ is communicated in the same way as the messages for Tx/Rx pairs in group $G_{3}$, whereas $M_{D_{\mathrm{Rx}}+2D_{\mathrm{Tx}}+2}^{\left(S,4\right)}$ is communicated in the same way as the messages for Tx/Rx pairs in group $G_{4}$.

*Group $G_{1}≜\left\{1,\ldots ,D_{Rx}+1\right\}$:* Each Tx $k\in G_{1}$ encodes its “slow” message $M_{k}^{\left(S\right)}$ using a codeword $X_{k}^{n}\left(M_{k}^{\left(S\right)}\right)$ from a Gaussian point-to-point code of power P, and transmits this codeword over the channel: $X_{k}^{n}=X_{k}^{n}\left(M_{k}^{\left(S\right)}\right)$. Each Rx $k\in G_{1}$ uses the cooperation message received from its left neighbour Rx k−1 for SIC, i.e., to delete the interference term $H_{k-1,k}X_{k-1}^{n}\left({\hat{M}}_{k-1}^{\left(S\right)}\right)$ from its output sequence $Y_{k}^{n}$:

(61) $${\hat{Y}}_{k}^{n}=Y_{k}^{n}-H_{k-1,k}X_{k-1}^{n}\left({\hat{M}}_{k-1}^{\left(S\right)}\right),$$

and to decode its desired message $M_{k}^{\left(S\right)}$ based on ${\hat{Y}}_{k}^{n}$. Rx *k* also describes its decoded message ${\hat{M}}_{k}$ over the cooperation link to Rx k+1, so as to facilitate SIC at this next Rx.

To facilitate the transmissions in the next group, the last Tx of group $G_{1}$, Tx $D_{\mathrm{Rx}}+1$, precodes its channel inputs $X_{D_{\mathrm{Rx}}+1}^{n}$ with the matrix $H_{D_{\mathrm{Rx}}+2,D_{\mathrm{Rx}}+2}^{-1}H_{D_{\mathrm{Rx}}+1,D_{\mathrm{Rx}}+2}$, quantises the produced sequence $I_{D_{\mathrm{Rx}}+1}^{n}≜H_{D_{\mathrm{Rx}}+2,D_{\mathrm{Rx}}+2}^{-1}H_{D_{\mathrm{Rx}}+1,D_{\mathrm{Rx}}+2}X_{D_{\mathrm{Rx}}+1}^{n}$ with a rate-L/2log(1+P) quantiser to obtain the quantisation ${\hat{I}}_{D_{\mathrm{Rx}}+1}^{n}$ at noise level and sends the resulting quantisation index as a first-round cooperation message to the first Tx in group $G_{2}$, i.e., to Tx $D_{\mathrm{Rx}}+2$.

*Group $G_{2}≜\left\{D_{Rx}+2,\ldots ,D_{Rx}+D_{Tx}+1\right\}$:* Each Tx $k\in G_{2}$ obtains a cooperation message from its left neighbour Tx k−1 that describes the quantised version ${\hat{I}}_{k-1}^{n}$ of $I_{k-1}^{n}≜H_{k,k}^{-1}H_{k-1,k}X_{k-1}^{n}$. Based on this message, Tx *k* reconstructs ${\hat{I}}_{k-1}^{n}$, encodes its “slow” message $M_{k}^{\left(S\right)}$ using a power P DPC that mitigates the interference ${\hat{I}}_{k-1}^{n}$, and sends the resulting DPC sequence $X_{k}^{n}$ over the channel. Moreover, it precodes this input sequence with the matrix $H_{k+1,k+1}^{-1}H_{k,k+1}$, quantises the precoded sequence $I_{k}^{n}≜H_{k+1,k+1}^{-1}H_{k,k+1}X_{k}^{n}$ with a rate-L/2log(1+P) quantiser (for a quantisation at noise level) to obtain ${\hat{I}}_{k}^{n}$, and sends the quantisation message as a round-*k* cooperation message over the link to its right neighbour. Tx $D_{\mathrm{Tx}}+D_{\mathrm{Rx}}+1$ produces its inputs in a similar way, i.e., using DPC, but sends no cooperation message at all. Rxs in $G_{2}$ use a standard DPC decoding rule based on the premultiplied outputs

(62) $$H_{k,k}^{-1}Y_{k}^{n}=H_{k,k}^{-1}H_{k-1,k}X_{k-1}^{n}+X_{k}^{n}+H_{k,k}^{-1}Z_{k}^{n},$$

to decode their intended “slow” messages. (Recall that $X_{k}^{n}$ was produced as a DPC sequence that mitigates ${\hat{I}}_{k-1}^{n}$, a quantised version of $H_{k,k}^{-1}H_{k-1,k}{\hat{X}}_{k-1}^{n}$). Since quantisation was performed at noise level, each message $M_{D_{\mathrm{Rx}}+2}^{\left(S\right)},\ldots ,M_{D+1}^{\left(S\right)}$ can be sent reliably with MG L.

*Group $G_{3}≜\left\{D_{Rx}+D_{Tx}+2,\ldots ,D_{Rx}+2D_{Tx}+2\right\}$:* This group of Tx/Rx pairs participates in the transmission of the “slow” messages

(63) $$M_{D_{\mathrm{Rx}}+D_{\mathrm{Tx}}+3}^{\left(S\right)},\ldots ,M_{D_{\mathrm{Rx}}+2D_{\mathrm{Tx}}+1}^{\left(S\right)},M_{D_{\mathrm{Rx}}+2D_{\mathrm{Tx}}+2}^{\left(S,3\right)}.$$

In particular, Tx $D_{\mathrm{Rx}}+2D_{\mathrm{Tx}}+2$ does not send an own message to its corresponding Rx.

Each of the messages in (63) is transmitted over the communication path Txk→Txk−1→Rxk for some $k\in \{D_{\mathrm{Rx}}+D_{\mathrm{Tx}}+3,\ldots ,D_{\mathrm{Rx}}+2D_{\mathrm{Tx}}+2\}$.

For each $k\in \{D_{\mathrm{Rx}}+D_{\mathrm{Tx}}+3,\ldots ,D_{\mathrm{Rx}}+2D_{\mathrm{Tx}}+2\}$, Tx *k* encodes its own “slow” message $M_{k}^{\left(S\right)}$ by means of DPC of power P that mitigates the interference $H_{k-1,k}^{-1}H_{k,k}X_{k}^{n}$ of the signal sent by Tx *k* itself; precodes the obtained sequence $U_{k}^{n}$ with the matrix $H_{k-1,k}^{-1}H_{k,k}$; quantises the precoded sequence $S_{k-1}^{n}≜H_{k-1,k}^{-1}H_{k,k}U_{k}^{n}$ to obtain a quantisation ${\hat{S}}_{k-1}$ at noise level; and sends the corresponding quantisation message as a (2D+3−k)-round cooperation message over the link to Tx k−1. Tx k−1 then reconstructs ${\hat{S}}_{k-1}^{n}$ and sends it over the channel: $X_{k-1}^{n}={\hat{S}}_{k-1}^{n}$. The construction of the transmit signal $X_{D_{\mathrm{Rx}}+2D_{\mathrm{Tx}}+2}^{n}$ mentioned above, is explained in the following paragraph. RXs $D_{\mathrm{Rx}}+D_{\mathrm{Tx}}+3,\ldots ,D_{\mathrm{Rx}}+2D_{\mathrm{Tx}}+2$ decode their intended “slow” messages using an optimal DPC decoding rule based on the premultiplied outputs

(64) $$H_{k-1,k}^{-1}Y_{k}^{n}=X_{k-1}^{n}+H_{k-1,k}^{-1}H_{k,k}X_{k}^{n}+H_{k-1,k}^{-1}Z_{k}^{n}.$$

Recall that $X_{k-1}^{n}$ is a quantised version (at noise level) of the precoded signal $S_{k-1}^{n}≜H_{k-1,k}^{-1}H_{k,k}U_{k}^{n}$ for $U_{k}^{n}$ a DPC sequence that mitigates the interference $H_{k-1,k}^{-1}H_{k,k}X_{k}^{n}$. Each of the messages $M_{D+3}^{\left(S\right)},\ldots ,M_{2D+2}^{\left(S\right)}$ can thus be transmitted reliably at full MG L.

*Group $G_{4}≜\left\{D_{Rx}+2D_{Tx}+2,\ldots ,2D_{Rx}+2D_{Tx}+2\right\}$:* This group of Tx/Rx pairs participates in the transmission of the “slow” messages

(65) $$M_{D_{\mathrm{Rx}}+2D_{\mathrm{Tx}}+2}^{\left(S,4\right)},M_{D_{\mathrm{Rx}}+2D_{\mathrm{Tx}}+3}^{\left(S\right)},\ldots ,M_{2D_{\mathrm{Rx}}+2D_{\mathrm{Tx}}+1}^{\left(S\right)}.$$

Tx $2D_{\mathrm{Rx}}+2D_{\mathrm{Tx}}+2$ thus is not sending an own message to its corresponding Rx. The messages in (65) are transmitted over the path Txk→Rxk+1→Rxk, for some $k\in \{D_{\mathrm{Rx}}+2D_{\mathrm{Tx}}+2,\ldots ,2D_{\mathrm{Rx}}+2D_{\mathrm{Tx}}+2\}$.

Each Tx $k\in \{D_{\mathrm{Rx}}+2D_{\mathrm{Tx}}+2,\ldots ,2D_{\mathrm{Rx}}+2D_{\mathrm{Tx}}+1\}$ encodes its “slow” message $M_{k}^{\left(S\right)}$ (or $M_{k}^{\left(S,4\right)}$ if $k=D_{\mathrm{Rx}}+2D_{\mathrm{Tx}}+2$) using a codeword from a Gaussian codebook of power P, and sends this codeword over the channel $X_{k}^{n}=X_{k}^{n}\left(M_{k}^{\left(S\right)}\right)$ (or $X_{k}^{n}=X_{k}^{n}\left(M_{k}^{\left(S,4\right)}\right)$ if $k=D_{\mathrm{Rx}}+2D_{\mathrm{Tx}}+1$).

Rx $2D_{\mathrm{Rx}}+2D_{\mathrm{Tx}}+2$ decodes $M_{2D_{\mathrm{Rx}}+2D_{\mathrm{Tx}}+1}^{\left(S\right)}$ based on an interference-free output $Y_{2D_{\mathrm{Rx}}+2D_{\mathrm{Tx}}+2}^{n}=H_{2D_{\mathrm{Rx}}+2D_{\mathrm{Tx}}+1,2D_{\mathrm{Rx}}+2D_{\mathrm{Tx}}+2}X_{2D_{\mathrm{Rx}}+2D_{\mathrm{Tx}}+1}^{n}+Z_{2D_{\mathrm{Rx}}+2D_{\mathrm{Tx}}+2}^{n}$, and sends the decoded message ${\hat{M}}_{2D_{\mathrm{Rx}}+2D_{\mathrm{Tx}}+1}^{\left(S\right)}$ over the cooperation link to the intended Rx $2D_{\mathrm{Rx}}+2D_{\mathrm{Tx}}+1$. For $k=2D_{\mathrm{Rx}}+2D_{\mathrm{Tx}}+1,\ldots ,D_{\mathrm{Rx}}+2D_{\mathrm{Tx}}+3$, Rx *k* uses the cooperation message received from its right neighbour Rx k+1 to decode $M_{k-1}^{\left(S\right)}$ (or $M_{k-1}^{\left(S,4\right)}$ if $k=D_{\mathrm{Rx}}+2D_{\mathrm{Tx}}+2$) using SIC, i.e., to first delete the interference $H_{k,k}X_{k}^{n}$ from $Y_{k}^{n}$ and then decode message $M_{k-1}^{\left(S\right)}$ (or $M_{k-1}^{\left(S,4\right)}$ if $k=D_{\mathrm{Rx}}+2D_{\mathrm{Tx}}+2$) from an interference-free signal. Rx *k* then sends the decoded message ${\hat{M}}_{k-1}^{\left(S\right)}$ (or ${\hat{M}}_{k-1}^{\left(S,4\right)}$ if $k=D_{\mathrm{Rx}}+2D_{\mathrm{Tx}}+2$) over the cooperation link to its left neighbour Rx k−1, which is the intended Rx for this message.

In the described scheme, each transmitted message is either decoded based on interference-free outputs or using DPC. Since precoding matrices do not depend on the power and quantizations are performed at noise levels, all messages can be transmitted reliably at MG L. Tx $D_{\mathrm{Rx}}+2D_{\mathrm{Tx}}+2$ sends two “slow” messages and $2D_{\mathrm{Rx}}+2D_{\mathrm{Tx}}-1$ other Txs send one “slow” message. An average “slow” MG of $L\cdot \frac{2D_{\mathrm{Rx}}+2D_{\mathrm{Tx}}+1}{2D_{\mathrm{Rx}}+2D_{\mathrm{Tx}}+2}$ is thus achieved in each subnet. Moreover, $2D_{\mathrm{Rx}}+2D_{\mathrm{Tx}}$ cooperation messages of prelog L are sent in each subnet:

1. Rxs in $G_{1}$ send $D_{\mathrm{Rx}}$ Rx-cooperation messages with prelog L;
2. Txs in $G_{2}$ send $D_{\mathrm{Tx}}$ Tx-cooperation messages with prelog L;
3. Txs in $G_{3}$ send $D_{\mathrm{Tx}}$ Tx-cooperation messages with prelog L;
4. Rxs in $G_{4}$ send $D_{\mathrm{Rx}}$ Rx-cooperation messages with prelog L.

The *average* cooperation prelog *per link* at the Tx-side is $\mu_{\mathrm{Tx},L}$ and at the Rx-side it is $\mu_{\mathrm{Rx},L}$. If one time-shares 2D+2 different instances of the described scheme with a different subset of silenced users in each of them, the overall scheme still achieves the MG pair $\left(S^{\left(F\right)}=0,S^{\left(S\right)}=L\frac{2D+1}{2D+2}\right)$ in (31d), each Tx-cooperation link is loaded at exactly this average cooperation prelog $\mu_{\mathrm{Tx},L}$, and each Rx-cooperation link is loaded at the average cooperation prelog $\mu_{\mathrm{Rx},L}$.

### 4.3. Scheme Achieving MG Pair (31d)

Consider the scheme described in the previous Section 4.2 and depicted in Figure 11. Notice that the first Tx in each subnet does not at all participate in the cooperation, and decoding of its message also does not rely on cooperation messages. The same observation applies also to the $D_{\mathrm{Rx}}+D_{\mathrm{Tx}}+1$st Tx of each subnet and its message. The first and the $D_{\mathrm{Rx}}+D_{\mathrm{Tx}}+1$st message of each subnet (the red Txs in Figure 11) thus satisfy the requirements on “fast” messages. We propose to use this scheme but let the first and the $\left(D_{\mathrm{Rx}}+D_{\mathrm{Tx}}+1\right)$st messages in each subnet be “fast” messages and all other messages be “slow” messages. This achieves the MG pair (31d).

The required cooperation rates equal $\mu_{\mathrm{Tx},L}$ and $\mu_{\mathrm{Rx},L}$, as explained in the previous Section 4.2.

### 4.4. Schemes Achieving MG Pair (31e)

**Figure 12:**
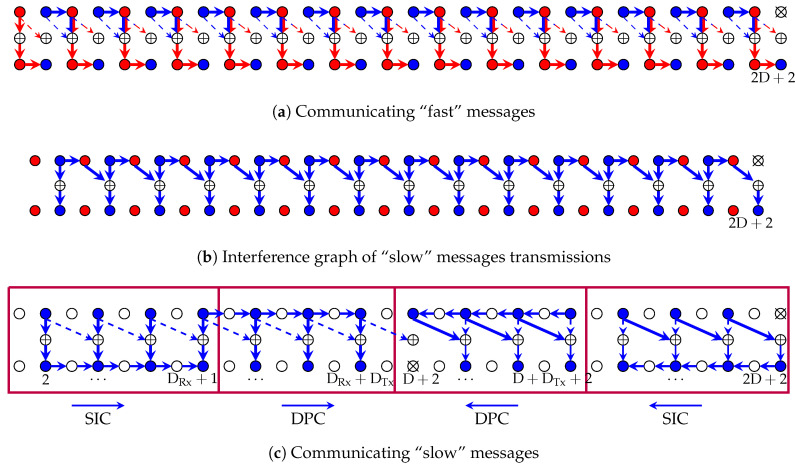
An illustration of the scheme achieving MG pair (31e). Notice that since D is even, the last Tx of $G_{2}$ sends a “fast” message. And since $D_{\mathrm{Rx}}$ is odd, also the first Tx in $G_{4}$ sends a “fast” message.

We periodically silence every 2D+2-nd Tx to split the network into smaller subnets. Then we send a “fast” message on all odd Txs and a “slow” message on all even Txs, except for the previously silenced Txs (which are all even). See Figure 12.

In what follows, we describe and analyze transmissions over the first subnet. Other subnets are treated analogously.

*Odd Txs 1,3,5,…,2D+1:* Each odd Tx encodes its “fast” message $M_{k}^{\left(F\right)}$ using a codeword $U_{k}^{n}\left(M_{k}^{\left(F\right)}\right)$ from a Gaussian codebook of power P that depends on the Tx and the channel realizations and is explained later. Tx 1 simply sends this Gaussian codeword $X_{1}^{n}=U_{1}^{n}\left(M_{1}^{\left(F\right)}\right)$. Any other odd Tx *k* first considers the cooperation message it received from its left neighbour Tx k−1 and reconstructs ${\hat{X}}_{k-1}^{n}$, a quantised version of Tx k−1th input $X_{k-1}^{n}$. Tx *k* then sends the input signal

(66) $$X_{k}^{n}=U_{k}^{n}\left(M_{k}^{\left(F\right)}\right)-H_{k,k}^{-1}H_{k-1,k}{\hat{X}}_{k-1}^{n}.$$

Odd Txs relay some of the cooperation messages they obtain from their neighbours, as will become clear in the following, but they do not create new cooperation messages.

*Odd Rxs 1,3,5,…,2D+1:* Given the precanceling at odd Txs described above, each odd Rx *k* observes an almost interference-free signal:

(67) $$Y_{k}^{n}=H_{k,k}U_{k}^{n}+H_{k-1,k}\left(X_{k-1}^{n}-{\hat{X}}_{k-1}^{n}\right)+Z_{k,k},$$

where notice that ${\hat{X}}_{k-1}^{n}$ is a quantised version of $X_{k-1}^{n}$ at noise level. Each odd Rx *k* therefore decodes its desired fast message $M_{k}^{\left(F\right)}$ using standard point-to-point decoding. It also sends the decoded message ${\hat{M}}_{k}^{\left(F\right)}$ over the cooperation link to its right neighbour Rx k+1 as a first round cooperation message.

Odd Rxs also relay some of the cooperation messages they obtain from their neighbours, as will become clear in the following.

Before describing the operations at the even Tx/Rx pairs, we make the following observations based on the operations at the odd Tx/Rx pairs. Irrespective of the operations performed at the even Txs, each even Rx *k* observes the sum of a signal depending only on “slow” messages and a signal depending only on its left-neighbour’s “fast” message (the signal $H_{k-1,k}U_{k-1}^{n}$). Since odd Rxs convey their decoded “fast” messages to their right-neighbour, even Rxs can cancel the signals depending on “fast” messages whenever they have been decoded correctly. There is thus no loss in reliable communication rate caused by the transmission of “fast” messages. And transmission of “slow” messages at even Txs can be designed as if no “fast” messages were present. However, if “slow” Rxs wish to send cooperation messages that do not depend on the “fast” transmissions, they have to wait for the second round.

*Even Txs 2,4,6,…,2D:* Each even Tx *k*, for k=2,…,2D, performs the same steps as Tx *k* in the scheme described in Section 4.2, but where the scheme needs to be adapted to include only even Txs. In particular, if an even Tx *k* previously sent a quantisation message to its direct left- or right-neighbour Tx k−1 or k+1, now it will send it to the previous or following *even* Tx k−2 or Tx k+2. (This simply means that the odd Tx lying between them has to relay the cooperation message as we already mentioned previously.) Similarly, when using DPC, if Tx *k* previously mitigated the quantised sequence ${\hat{I}}_{k-1}^{n}$ or ${\hat{S}}_{k+1}^{n}$, now it mitigates the quantised sequence ${\hat{I}}_{k-2}^{n}$ or ${\hat{S}}_{k+2}^{n}$. Notice that since D is even, Tx D is the last even Tx in $G_{2}$ (so the last Tx in $G_{2}$ sending a “slow” message). Tx-cooperation in group $G_{2}$ thus takes place only during the first $D_{\mathrm{Tx}}-1$ rounds. The only Tx-cooperation message in round $D_{\mathrm{Tx}}$ is the message sent from Tx D+3 to Tx D+2 in group $G_{3}$.

In addition, if this is not already done as part of the scheme in Section 4.2, any even Tx *k* also quantizes its channel inputs $X_{k}^{n}$ at rate L·1/2log(1+P) to generate the quantised sequence ${\hat{I}}_{k}^{n}$. The quantisation message describing ${\hat{I}}_{k}^{n}$ is then sent as a $D_{\mathrm{Tx}}$-round cooperation message over the link to Tx k+1 to allow this Tx to precancel this interference in the way that was described previously. Even Tx D+2 (the first Tx in group $G_{3}$) does not need to send this round-$D_{\mathrm{Tx}}$ cooperation message because its right neighbour Tx D+3 already learns the Tx signal $X_{D+2}^{n}$ as part of the proposed scheme in Section 4.2. Since all even Txs (except for Tx D+2) receive their last cooperation message in round $D_{\mathrm{Tx}}-1$, they can indeed compute their input perior to the last round $D_{\mathrm{Tx}}$ and thus perform the proposed round-$D_{\mathrm{Tx}}$ cooperation.

*Even Rxs 2,4,6,…,2D+2:* Using the round-1 Rx-cooperation messages from its left neighbour, each even Rx *k*, for k=2,…,2D+2, first subtracts the interference caused by the transmission of the “fast” message $M_{k-1}^{\left(F\right)}$ at its left neighbour. That means, it forms

(68) $$\begin{array}{c} {\tilde{Y}}_{k}^{n}=Y_{k}^{n}-H_{k-1,k}U_{k-1}^{n}. \end{array}$$

It then proceeds with this modified output sequence ${\tilde{Y}}_{k}^{n}$ and performs all the steps as Rx *k* did in the scheme in Section 4.2, but where the scheme again needs to be adapted to include only even Rxs and it also needs to be adapted to start only at cooperation round 2. This allows even Rxs to calculate (68) before performing the other steps. Notice that since the first Txs of $G_{1}$ and $G_{4}$ only send “fast” messages (the latter holds because $D_{\mathrm{Rx}}$ is odd), there is no harm in waiting for this second round. To adapt the scheme in Section 4.2 only to even Rxs, any even Rx *k* that previously sent its decoded message to its direct left- or right-neighbour Rx k−1 or Rx k+1, now sends it to the previous or following *even* Rx k−2 or Rx k+2. Similarly, any Rx *k* that previously applied the SIC step to cancel the interference from Tx k−1 or Tx k+1, now cancels the interference from Tx k−2 or Tx k+2.

In the described scheme, all odd Txs of a subnet can send reliably a “fast” message of MG L and the even Txs {2,4,…,2D} each can send reliably a “slow” message of MG L. The scheme thus achieves the MG pair in (31e): $\left(S^{\left(F\right)}=\frac{L}{2},S^{\left(S\right)}=L\cdot \frac{D}{2D+2}\right)$.

We now analyze the cooperation prelog of the described scheme. Recall that in this scheme each even Tx sends a quantised version of its inputs to its right neighbour and each odd Rx sends its decoded message to its right neighbour. Since each of these cooperation messages is of prelog L the described messages consume a Tx-cooperation prelog of L·D and a Rx-cooperation prelog of L·D.

*In addition*, for encoding and decoding of “slow” messages:

1. Rxs in $G_{1}$ send $D_{\mathrm{Rx}}-1$ Rx-cooperation messages with prelog L. (The cooperation message from Rx 1 to Rx 2 has already been counted in the previous paragraph.)
2. Txs in $G_{2}$ send $(D_{\mathrm{Tx}}-1)/2$ Tx-cooperation messages with prelog L. (The cooperation message from even to odd Txs in $G_{2}$ have already been counted in the previous paragraph.)
3. Txs in $G_{3}$ send $D_{\mathrm{Tx}}$ Tx-cooperation messages with prelog L.
4. Rxs in $G_{4}$ send $D_{\mathrm{Rx}}-1$ Rx-cooperation messages with prelog L. (The first Rx in $G_{4}$ does not obtain a cooperation message because it is a “fast” Tx.)

To summarize, the described scheme requires an *average prelog per Tx-cooperation link* of $\mu_{\mathrm{Tx},H}=L\cdot \frac{\frac{D}{2}+\frac{3}{4}D_{\mathrm{Tx}}-\frac{1}{4}}{2D+2}$ and an *average prelog per Rx-cooperation link* of $\mu_{\mathrm{Rx},H}=L\cdot \frac{\frac{D}{2}+D_{\mathrm{Rx}}-1}{2D+2}$. (Notice that this is larger than in the scheme in Section 4.2.) If one time-shares 2D+2 different instances of the described scheme with a different subset of silenced users in each of them, the required prelog on each Tx-cooperation link is exactly $\mu_{\mathrm{Tx},H}$ and the required prelog on each Rx-cooperation link is exactly $\mu_{\mathrm{Rx},H}$. This concludes the proof.

## 5. Summary and Concluding Remarks

We considered Wyner’s soft-handoff network and characterized the MG region with transmitter and receiver cooperation when part of the messages are subject to stringent delay constraints. For the setup with *only* transmitter or *only* receiver cooperation we observed the following. Increasing the MG of delay-sensitive messages by Δ requires decreasing the MG of delay-tolerant messages approximately by 2Δ. This penalty does not arise when both transmitters *and* receivers can cooperate. More precisely, for small cooperation prelogs, when delay-sensitive messages have moderate or small MGs, then the *sum*-MG is not decreased compared to when only delay-tolerant messages are transmitted. For large cooperation prelogs, this conclusion even holds when delay-sensitive messages have large MGs.

An interesting line of future work concerns extending the existing results to two-dimensional cellular models (i.e., to models where transmitters and receivers are not aligned on a grid). First results on the hexagonal Wyner model [18] indicate that similar conclusions hold as for Wyner’s soft-handoff model investigated in this talk. Another interesting line of future work studies the impact of channel state information (CSI) at the transmitter as in Reference [19] but for the considered model with mixed-delay constraints. In particular a model where CSI is present for en/decoding delay-tolerant messages but not for en/decoding of delay-sensitive messages is a natural extension of the presented setup.

## Appendix A. Proof of the Converse to (15)

For convenience of notation, define for any k∈{1,…,K}:

(A1) $$M_{k}≜\left(M_{k}^{\left(F\right)},M_{k}^{\left(S\right)}\right).$$

For each power P>0, fix a sequence (in the blocklength *n*) of encoding and decoding functions respecting the power constraints and the Rx-cooperation rate-limitations (recall that we consider a setup with only Rx-cooperation but no Tx-cooperation) such that the error probability p(error)→0 as the blocklength n→∞.

By Fano’s Inequality there exists a sequence $\epsilon_{n}$ satisfying $\frac{\epsilon_{n}}{n}\to 0$ as n→∞ such that for any k∈[1:K−1] and each blocklength *n*:

(A2) $$\begin{array}{ccc} R_{k}^{\left(F\right)} & + & R_{k}^{\left(S\right)}+R_{k+1}^{\left(F\right)}\;\\ & = & \frac{1}{n}\left[H\left(M_{k}^{\left(F\right)}\right)+H\left(M_{k}^{\left(S\right)}\right)+H\left(M_{k+1}^{\left(F\right)}\right)\right]\\ & = & \;\frac{1}{n}[H\left(M_{k}^{\left(F\right)}|M_{k-1}\right) \end{array}$$

(A3) $$\begin{array}{cc} & \;+H\left(M_{k}^{\left(S\right)}|M_{1},\ldots ,M_{k-1},M_{k}^{\left(F\right)},M_{k+1},\ldots ,M_{K}\right)+H\left(M_{k+1}^{\left(F\right)}|M_{k-1},M_{k+1}^{\left(S\right)}\right)]\\ \leq & \;\frac{1}{n}[I\left(M_{k}^{\left(F\right)};Y_{k}^{n}|M_{k-1}\right)+I\left(M_{k}^{\left(S\right)};Y_{1}^{n},\ldots ,Y_{K}^{n}|M_{1},\ldots ,M_{k-1},M_{k}^{\left(F\right)},M_{k+1},\ldots ,M_{K}\right) \end{array}$$

(A4) $$\begin{array}{cc} & \;+I\left(M_{k+1}^{\left(F\right)};Y_{k+1}^{n}|M_{k-1},M_{k+1}^{\left(S\right)}\right)]+\frac{\epsilon_{n}}{n}\\ \overset{\left(a\right)}{=} & \frac{1}{n}[I\left(M_{k}^{\left(F\right)};Y_{k}^{n}|M_{k-1}\right)+I\left(M_{k}^{\left(S\right)};Y_{k}^{n},Y_{k+1}^{n}|M_{k-1},M_{k}^{\left(F\right)},M_{k+1}\right) \end{array}$$

(A5) $$\begin{array}{cc} & \;+I\left(M_{k+1}^{\left(F\right)};Y_{k+1}^{n}|M_{k-1},M_{k+1}^{\left(S\right)}\right)]+\frac{\epsilon_{n}}{n}\\ \overset{\left(b\right)}{=} & \frac{1}{n}[I\left(M_{k}^{\left(F\right)},M_{k}^{\left(S\right)};Y_{k}^{n}|M_{k-1}\right)+I\left(M_{k}^{\left(S\right)};Y_{k+1}^{n}|Y_{k}^{n},M_{k}^{\left(F\right)},M_{k-1},M_{k+1}\right) \end{array}$$

(A6) $$\begin{array}{cc} & \;\;+I\left(M_{k+1}^{\left(F\right)};Y_{k+1}^{n}|M_{k-1},M_{k+1}^{\left(S\right)}\right)]+\frac{\epsilon_{n}}{n}\\ \leq & \;\frac{1}{n}[h\left(H_{k,k}X_{k}^{n}+Z_{k}^{n}\right)-h\left(Z_{k}^{n}\right)+h\left(H_{k,k+1}X_{k}^{n}+Z_{k+1}^{n}|H_{k,k}X_{k}^{n}+Z_{k}^{n}\right) \end{array}$$

(A7) $$\begin{array}{cc} & \;\;-h\left(Z_{k+1}^{n}\right)+h\left(Y_{k+1}^{n}|M_{k+1}^{\left(S\right)}\right)-h\left(H_{k,k+1}X_{k}^{n}+Z_{k+1}^{n}\right)]+\frac{\epsilon_{n}}{n}\\ \overset{\left(c\right)}{\leq } & \sum_{i=1}^{L}\frac{1}{2}\log\left(1+{\left(\sum_{j=1}^{L}\left|H_{k+1,k+1}\left(i,j\right)\right|\right)}^{2}P+{\left(\sum_{j=1}^{L}\left|H_{k,k+1}\left(i,j\right)\right|\right)}^{2}P\right)\\ & +\frac{1}{2}\log\det\left(I_{L}+H_{k,k+1}{H_{k,k}}^{-1}{H_{k,k}}^{-T}H_{k,k+1}^{T}\right) \end{array}$$

(A8) $$\begin{array}{c} +\frac{1}{2}\log\det\left(H_{k,k}^{-1}H_{k,k}^{-T}+H_{k,k+1}^{-1}H_{k,k+1}^{-T}\right)+\log\det\left(H_{k,k}\right)+\frac{\epsilon_{n}}{n}, \end{array}$$

where $I_{L}$ denotes the L-by-L identity matrix and $H_{k+1,k+1}\left(i,j\right)$ and $H_{k,k+1}\left(i,j\right)$ denote the elements of matrices $H_{k+1,k+1}$ and $H_{k,k+1}$ in row *i* and column *j*. Here, (a) follows because given source messages $M_{k-1}$ and $M_{k+1}$, the triple $\left(M_{k},Y_{k}^{n},Y_{k+1}^{n}\right)$ is independent of the rest of the outputs $Y_{1}^{n},\ldots ,Y_{k-1}^{n},Y_{k+2}^{n},\ldots ,Y_{K}^{n}$ and source messages $M_{1},\ldots ,M_{k-2},M_{k+2},\ldots ,M_{K}$; (b) follows by the chain rule of mutual information and because $M_{k+1}$ is independent of the tuple $\left(M_{k-1},M_{k},Y_{k}^{n}\right)$; and (c) is obtained by rearranging terms, and the following bounds (A12), (A15) and (A24).

We first bound the term $h(Y_{k+1}^{n}|M_{k+1}^{\left(S\right)})$, and start by noting that because conditioning can only reduce entropy and by the entropy-maximizing property of the Gaussian distribution:

(A9) $$\begin{array}{ccc} h(Y_{k+1}^{n}|M_{k+1}^{\left(S\right)}) & \leq & \sum_{i=1}^{L}\sum_{t=1}^{n}h\left(Y_{k+1,t}\left(i\right)\right)\leq \sum_{i=1}^{L}\sum_{t=1}^{n}\frac{1}{2}\log\left(\left(2\pi e\right)\mathrm{Var}\left(Y_{k+1,t}\left(i\right)\right)\right), \end{array}$$

where $Y_{k+1,t}\left(i\right)$ denotes the *i*-th entry of the vector $Y_{k+1,t}$. Recall that in this setup without Tx-cooperation the input vectors $X_{k}^{n}$ and $X_{k+1}^{n}$ are independent. However the elements of each input vector can be arbitrarily correlated. The variance $\mathrm{Var}(Y_{k+1,t}\left(i\right))$ is maximized if the elements of $X_{k+1,t}$ are fully correlated and thus:

(A10) $$\mathrm{Var}\left(Y_{k+1,t}\left(i\right)\right)\leq 1+{\left(\sum_{j=1}^{L}\left|H_{k,k+1}\left(i,j\right)\right|\sqrt{P_{k,t}\left(j\right)}\right)}^{2}+{\left(\sum_{j=1}^{L}\left|H_{k+1,k+1}\left(i,j\right)\right|\sqrt{P_{k+1,t}\left(j\right)}\right)}^{2},$$

where $P_{k,t}\left(j\right)$ and $P_{k+1,t}\left(j\right)$ denote the variances of the *j*-th elements of input vectors $X_{k,t}^{n}$ and $X_{k+1,t}^{n}$. In the following we relax the power constraint (7) by requiring only that the power of the *n* channel inputs produced by any given Tx-antenna cannot exceed nP:

(A11) $$\sum_{t=1}^{n}P_{k,t}\left(j\right)\leq nP,\;k\in \left\{1,\ldots ,K\right\}\;\mathrm{and}\;j\in \left\{1,\ldots ,L\right\}.$$

Since the right-hand side of (A10) is monotonically increasing and jointly concave in the powers $\left\{P_{k,t}\left(j\right)\right\}$ and $\left\{P_{k+1,t}\left(j\right)\right\}$, the upper bound on $\mathrm{Var}(Y_{k+1,t}\left(i\right))$ is largest when $P_{k,t}\left(j\right)=P_{k+1,t}\left(j\right)=P$. Moreover since also the function x↦log(1+x) is monotonically increasing, we conclude:

(A12) $$\begin{array}{ccc} h(Y_{k+1}^{n}|M_{k+1}^{\left(S\right)}) & \leq & \;n\sum_{i=1}^{L}\frac{1}{2}\log\left(2\pi e\right)\left(1+{\left(\sum_{j=1}^{L}\left|H_{k,k+1}\left(i,j\right)\right|\right)}^{2}P+{\left(\sum_{j=1}^{L}\left|H_{k+1,k+1}\left(i,j\right)\right|\right)}^{2}P\right). \end{array}$$

We next bound the term

(A13) $$\begin{array}{ccc} \frac{1}{n}h\left(H_{k,k+1}X_{k}^{n}+Z_{k+1}^{n}|H_{k,k}X_{k}^{n}+Z_{k}^{n}\right) & = & \frac{1}{n}h\left(Z_{k+1}^{n}-H_{k,k+1}{H_{k,k}}^{-1}Z_{k}^{n}|H_{k,k}X_{k}^{n}+Z_{k}^{n}\right) \end{array}$$

(A14) $$\begin{array}{cc} \leq & \frac{1}{n}h\left(Z_{k+1}^{n}-H_{k,k+1}{H_{k,k}}^{-1}Z_{k}^{n}\right) \end{array}$$

(A15) $$\begin{array}{cc} = & \frac{1}{2}\log\det\left(I_{L}+H_{k,k+1}{H_{k,k}}^{-1}{H_{k,k}}^{-T}H_{k,k+1}^{T}\right), \end{array}$$

where recall that $I_{L}$ denotes the L-by-L identity matrix.

For the last bound, define

(A16) $$T_{k}^{n}≜H_{k,k}^{-1}Z_{k}^{n}-H_{k,k+1}^{-1}Z_{k+1}^{n}.$$

Then:

$$\begin{array}{c} \frac{1}{n}h\left(H_{k,k}X_{k}^{n}+Z_{k}^{n}\right)-\frac{1}{n}h\left(H_{k,k+1}X_{k}^{n}+Z_{k+1}^{n}\right)\; \end{array}$$

(A17) $$\begin{array}{cc} \overset{\left(e\right)}{=} & \frac{1}{n}h\left(X_{k}^{n}+H_{k,k+1}^{-1}Z_{k+1}^{n}+T_{k}^{n}\right)-\frac{1}{n}h\left(X_{k}^{n}+H_{k,k+1}^{-1}Z_{k+1}^{n}\right)+\log\left(\frac{\det(H_{k,k})}{\det(H_{k,k+1})}\right) \end{array}$$

(A18) $$\begin{array}{cc} \overset{\left(f\right)}{\leq } & \frac{1}{n}h\left(X_{k}^{n}+H_{k,k+1}^{-1}Z_{k+1}^{n}+T_{k}^{n}\right)-\frac{1}{n}h\left(X_{k}^{n}+H_{k,k+1}^{-1}Z_{k+1}^{n}|T_{k}^{n}\right)+\log\left(\frac{\det(H_{k,k})}{\det(H_{k,k+1})}\right) \end{array}$$

(A19) $$\begin{array}{cc} = & \frac{1}{n}I\left(X_{k}^{n}+H_{k,k+1}^{-1}Z_{k+1}^{n}+T_{k}^{n};T_{k}^{n}\right)+\log\left(\frac{\det(H_{k,k})}{\det(H_{k,k+1})}\right) \end{array}$$

(A20) $$\begin{array}{cc} \overset{\left(g\right)}{\leq } & \frac{1}{n}I\left(H_{k,k+1}^{-1}Z_{k+1}^{n}+T_{k}^{n};T_{k}^{n}|X_{k}^{n}\right)+\log\left(\frac{\det(H_{k,k})}{\det(H_{k,k+1})}\right) \end{array}$$

(A21) $$\begin{array}{cc} \overset{\left(g\right)}{=} & \frac{1}{n}I\left(H_{k,k+1}^{-1}Z_{k+1}^{n}+T_{k}^{n};T_{k}^{n}\right)+\log\left(\frac{\det(H_{k,k})}{\det(H_{k,k+1})}\right) \end{array}$$

(A22) $$\begin{array}{cc} \overset{\left(h\right)}{=} & \frac{1}{n}h\left(T_{k}^{n}\right)-h\left(H_{k,k+1}^{-1}Z_{k+1}^{n}\right)+\log\left(\frac{\det(H_{k,k})}{\det(H_{k,k+1})}\right) \end{array}$$

(A23) $$\begin{array}{cc} = & \frac{1}{2}\log\frac{\det\left(H_{k,k}^{-1}H_{k,k}^{-T}+H_{k,k+1}^{-1}H_{k,k+1}^{-T}\right)}{\det(H_{k,k+1}^{-1}H_{k,k+1}^{-T})}+\log\left(\frac{\det(H_{k,k})}{\det(H_{k,k+1})}\right) \end{array}$$

(A24) $$\begin{array}{cc} = & \frac{1}{2}\log\det\left(H_{k,k}^{-1}H_{k,k}^{-T}+H_{k,k+1}^{-1}H_{k,k+1}^{-T}\right)+\log\det\left(H_{k,k}\right), \end{array}$$

where (e) holds by the definition of $T_{k}^{n}$ and because h(AX)=logdet(A)+h(X) for any matrix A and vector X; (f) holds because conditioning can only reduce entropy; (inequalities) (g) hold again because conditioning can only reduce entropy and by the independence of $T_{k}^{n}$ and $X_{k}^{n}$; and (h) holds because by the independence of the noise vectors we have $h\left(T_{k}^{n}|H_{k,k+1}^{-1}Z_{k+1}^{n}+T_{k}^{n}\right)=h\left(-H_{k,k+1}^{-1}Z_{k}^{n}|H_{k,k}^{-1}Z_{k}^{n}\right)=h\left(H_{k,k+1}^{-1}Z_{k}^{n}\right)$.

Following similar steps as the ones leading to (A12), one can also prove that

(A25) $$\begin{array}{ccc} R_{1}^{\left(F\right)} & \leq & \frac{1}{n}I\left(M_{1}^{\left(F\right)};Y_{K}^{n}|M_{1}^{\left(S\right)}\right)+\frac{\epsilon_{n}}{n} \end{array}$$

(A26) $$\begin{array}{cc} \leq & \sum_{i=1}^{L}\frac{1}{2}\log\left(1+{\left(\sum_{j=1}^{L}\left|H_{1,1}\left(i,j\right)\right|\right)}^{2}P\right)+\frac{\epsilon_{n}}{n}, \end{array}$$

and

(A27) $$\begin{array}{ccc} R_{K}^{\left(F\right)}+R_{K}^{\left(S\right)} & \leq & \frac{1}{n}I\left(M_{K}^{\left(F\right)},M_{K}^{\left(S\right)};Y_{K}^{n}|M_{K-1}\right)+\frac{\epsilon_{n}}{n} \end{array}$$

(A28) $$\begin{array}{cc} \leq & \sum_{i=1}^{L}\frac{1}{2}\log\left(1+{\left(\sum_{j=1}^{L}\left|H_{K,K}\left(i,j\right)\right|\right)}^{2}P\right)+\frac{\epsilon_{n}}{n}, \end{array}$$

where $H_{1,1}\left(i,j\right)$ and $H_{K,K}\left(i,j\right)$ denote row-*i*, column-*j* elements of the matrices $H_{1,1}$ and $H_{K,K}$.

We sum up the bound in (A8) for all values of k∈{1,…,K−1}, and combine it with (A26) and (A28). Taking n→∞, it follows that because the probability of error p(error) vanishes as n→∞ (and thus $\frac{\epsilon_{n}}{n}\to 0$ as n→∞):

(A29) $$\begin{array}{c} \sum_{k=1}^{K}\left(2R_{k}^{\left(F\right)}+R_{k}^{\left(S\right)}\right)\\ \;=R_{1}^{\left(F\right)}+\sum_{k=1}^{K-1}\left(R_{k}^{\left(F\right)}+R_{k}^{\left(S\right)}+R_{k+1}^{\left(F\right)}\right)+R_{K}^{\left(F\right)}+R_{K}^{\left(S\right)}\\ \;\leq \sum_{k=1}^{K-1}[\sum_{i=1}^{L}\frac{1}{2}\log\left(1+{\left(\sum_{j=1}^{L}\left|H_{k+1,k+1}\left(i,j\right)\right|\right)}^{2}P+{\left(\sum_{j=1}^{L}\left|H_{k,k+1}\left(i,j\right)\right|\right)}^{2}P\right)\\ \;+\frac{1}{2}\log\det\left(I_{L}+H_{k,k+1}{H_{k,k}}^{-1}{H_{k,k}}^{-T}H_{k,k+1}^{T}\right)+\frac{1}{2}\log\det\left(H_{k,k}^{-1}H_{k,k}^{-T}+H_{k,k+1}^{-1}H_{k,k+1}^{-T}\right)+\log\det\left(H_{k,k}\right)] \end{array}$$

(A30) $$\begin{array}{c} +\sum_{i=1}^{L}\frac{1}{2}\log\left(1+{\left(\sum_{j=1}^{L}\left|H_{1,1}\left(i,j\right)\right|\right)}^{2}P\right)+\sum_{i=1}^{L}\frac{1}{2}\log\left(1+{\left(\sum_{j=1}^{L}\left|H_{K,K}\left(i,j\right)\right|\right)}^{2}P\right). \end{array}$$

Dividing by *K* and $\frac{1}{2}\log\left(P\right)$ and taking P,K→∞, establishes the converse bound (15).

## Appendix B. Proof of Converse to (21)

Fix a sequence (in the blocklength *n*) of encoding and decoding functions respecting the power constraints and the Tx-cooperation rate-limitations (recall that we consider a setup with only Tx-cooperation but no Rx-cooperation) such that the error probability p(error)→0 as the blocklength n→∞.

Let $M^{\left(S\right)}≜(M_{1}^{\left(S\right)},\ldots ,$$M_{K}^{\left(S\right)})$. By Fano’s Inequality there exists a sequence $\epsilon_{n}$ satisfying $\frac{\epsilon_{n}}{n}\to 0$ as n→∞ such that for any k∈{1,…,K−1} and any blocklength *n*:

(A31) $$\begin{array}{ccc} R_{k}^{\left(F\right)} & + & R_{k+1}^{\left(S\right)}+R_{k+1}^{\left(F\right)}\\ & = & \frac{1}{n}\left[H\left(M_{k}^{\left(F\right)}|M^{\left(S\right)},M_{k-1}^{\left(F\right)}\right)+H\left(M_{k+1}|M_{1}^{\left(S\right)},\ldots ,M_{k}^{\left(S\right)},M_{k+2}^{\left(S\right)},\ldots ,M_{K}^{\left(S\right)}\right)\right]+\frac{\epsilon_{n}}{n}\\ & \leq & \frac{1}{n}\left[I\left(M_{k}^{\left(F\right)};Y_{k}^{n}|M^{\left(S\right)},M_{k-1}^{\left(F\right)}\right)+I\left(M_{k+1};Y_{k+1}^{n}|M_{1}^{\left(S\right)},\ldots ,M_{k}^{\left(S\right)},M_{k+2}^{\left(S\right)},\ldots ,M_{K}^{\left(S\right)}\right)\right]+\frac{\epsilon_{n}}{n}\\ & = & \frac{1}{n}[h\left(H_{k,k}X_{k}^{n}+Z_{k}^{n}|M^{\left(S\right)}\right)-h\left(Z_{k}^{n}\right)\\ & & \;+h\left(Y_{k+1}^{n}|M_{1}^{\left(S\right)},\ldots ,M_{k}^{\left(S\right)},M_{k+2}^{\left(S\right)},\ldots ,M_{K}^{\left(S\right)}\right)-h\left(H_{k,k+1}X_{k}^{n}+Z_{k+1}^{n}|M^{\left(S\right)}\right)]+\frac{\epsilon_{n}}{n}\\ & \overset{\left(a\right)}{\leq } & \sum_{i=1}^{L}\frac{1}{2}\log\left(1+{\left(\sum_{j=1}^{L}\left(\right|H_{k+1,k+1}\left(i,j\right)|+|H_{k,k+1}\left(i,j\right)\left|\right)\right)}^{2}P\right)\\ & & +\frac{1}{2}\log\det\left(H_{k,k}^{-1}H_{k,k}^{-T}+H_{k,k+1}^{-1}H_{k,k+1}^{-T}\right)+\log\det\left(H_{k,k}\right)+\frac{\epsilon_{n}}{n}, \end{array}$$

where (a) follows by similar steps as lead to (A15) and (A24), but where one has to account for the fact that due to the Tx-cooperation, the input vectors $X_{k}^{n}$ and $X_{k+1}^{n}$ can be correlated.

Similarly to (A24) one can further prove that

(A32) $$R_{1}^{\left(F\right)}\leq \sum_{i=1}^{L}\frac{1}{2}\log\left(1+{\left(\sum_{j=1}^{L}\left|H_{1,1}\left(i,j\right)\right|\right)}^{2}P\right)+\frac{\epsilon_{n}}{n},$$

and

(A33) $$R_{K}^{\left(F\right)}+R_{K}^{\left(S\right)}\leq \sum_{i=1}^{L}\frac{1}{2}\log\left(1+{\left(\sum_{j=1}^{L}\left(\right|H_{K,K}\left(i,j\right)|+|H_{K-1,K}\left(i,j\right)\left|\right)\right)}^{2}P\right)+\frac{\epsilon_{n}}{n},$$

where again one has to consider that because of the Tx-cooperation the various input vectors can be correlated.

We now sum up the bound in (A31) for all values of k∈{1,…,K−1} and combine it with (A32) and (A33). Taking n→∞, it follows that because the probability of error p(error) vanishes as n→∞ (and thus $\frac{\epsilon_{n}}{n}\to 0$ as n→∞):

(A34) $$\begin{array}{c} \sum_{k=1}^{K}\left(2R_{k}^{\left(F\right)}+R_{k}^{\left(S\right)}\right)\\ \;=R_{1}^{\left(F\right)}+\sum_{k=1}^{K-1}\left(R_{k}^{\left(F\right)}+R_{k}^{\left(S\right)}+R_{k+1}^{\left(F\right)}\right)+R_{K}^{\left(F\right)}+R_{K}^{\left(S\right)})\\ \;\leq \sum_{k=1}^{K-1}[\sum_{i=1}^{L}\frac{1}{2}\log\left(1+{\left(\sum_{j=1}^{L}\left(\right|H_{k+1,k+1}\left(i,j\right)|+|H_{k,k+1}\left(i,j\right)\left|\right)\right)}^{2}P\right)\\ \;\;+\frac{1}{2}\log\det\left(H_{k,k}^{-1}H_{k,k}^{-T}+H_{k,k+1}^{-1}H_{k,k+1}^{-T}\right)+\log\det\left(H_{k,k}\right)]\\ \;+\sum_{i=1}^{L}\frac{1}{2}\log\left(1+{\left(\sum_{j=1}^{L}\left|H_{1,1}\left(i,j\right)\right|\right)}^{2}P\right)+\sum_{i=1}^{L}\frac{1}{2}\log\left(1+{\left(\sum_{j=1}^{L}\left(\right|H_{K,K}\left(i,j\right)|+|H_{K-1,K}\left(i,j\right)\left|\right)\right)}^{2}P\right). \end{array}$$

Dividing by *K* and $\frac{1}{2}\log\left(P\right)$ and taking P,K→∞, establishes the converse bound (21).
